# Incorporating Implicit Information to Disentangle the Impacts of Hydropower Dams and Climate Change on Basin‐Scale Fish Habitat Distribution

**DOI:** 10.1002/ece3.70412

**Published:** 2024-10-08

**Authors:** Xiongfeng Bai, Peng Zhang, Xin Cao, Dongya Zhang, Zhi Yang, Xianghong Dong, Siyang Wang, Wenbin Li, Lihua Xiong

**Affiliations:** ^1^ State Key Laboratory of Water Resources Engineering and Management Wuhan University Wuhan Hubei China; ^2^ Beijing Engineering Corporation Limited Power China Beijing China; ^3^ Institute of Hydroecology Chinese Academy of Science and Ministry of Water Resource Wuhan China; ^4^ College of Animal Science Guizhou University Guiyang China; ^5^ School of Civil Engineering, Architecture and Environment Hubei University of Technology Wuhan China

**Keywords:** climate change, dam impact, fish habitat, species distribution model, warm‐water and cold‐water fish

## Abstract

The loss of freshwater fish habitats, exacerbated by climate change and dam constructions, poses a critical environmental concern. The upper Yangtze River basin, noted for its abundant fish fauna and concentrated dam development, serves as a crucial locale for investigating the impacts of climate shifts and dam construction. This study aims to disentangle the impacts of hydroelectric dams and climate change on fish habitat distribution by analyzing species presence data across different periods. Species distribution models were constructed using Maxent for *Coreius guichenoti* (a warm‐water endangered fish) and *Schizopygopsis malacanthus* (a cold‐water endangered fish). The model accuracy was assessed using the area under the curve of the receiver operating characteristic. Habitat distribution modeling and prediction for the pre‐dam period (1970–2000) and post‐dam period (2001–2020), as well as future climate change under two shared socioeconomic pathways scenarios, were conducted. The impacts of climate change and dam construction on the habitat suitability of two fish species were quantified. The results revealed dam construction predominantly diminished habitat suitability and range, with high‐suitability habitats in the post‐dam period decreasing by 56.3% (720.18 km) and 67.0% (1665.52 km) for the two fishes, respectively. Climate change would enhance the habitat suitability of *Coreius guichenoti*, while it would decrease the habitat suitability of *Schizopygopsis malacanthus*. The impact of dam construction is greater that of climate change for them. This study underscores the profound impacts of dam construction on fish habitats, particularly for cold‐water species, and highlights the critical need for habitat restoration in sustainable hydropower development. Our method of disentangling these factors also provides a new approach to evaluating environmental impacts in large river basins.

## Graphical abstract

Understanding how cold‐water and warm‐water fish cope with various stressors such as climate change and dam construction is crucial for the conservation of freshwater fish ecosystems. This study proposes to incorporate the information on dam impact implicitly existing in the species presence data in different periods to disentangle the impacts of hydroelectric dams and climate change on cold‐water and warm‐water fish habitat distribution in the upper Yangtze River basin.

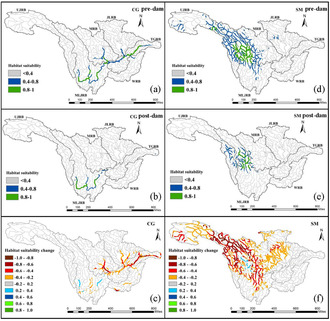

## 1 Introduction

Climate change and human activities are driving unprecedented biodiversity loss, marking the current sixth biodiversity crisis (Cardinale et al. 2012). Freshwater ecosystems are declining faster than even the most affected terrestrial ecosystems (Sala et al. 2000). Freshwater fishes, with over 10,000 species, represent about 40% of global fish populations and one‐quarter of vertebrates (Lundberg et al. 2000). Global climate change alters river flow regimes and increases water temperatures, impacting the habitat quality and availability for freshwater fishes (Zhang et al. 2019). These changes affect fish physiology and behavior, potentially leading to significant shifts in distribution and community diversity (Parmesan and Yohe 2003; Cheng et al. 2019). The degradation of the aquatic environment, the loss of suitable habitats, and biological invasions caused by human activities can also pose serious threats to freshwater fishes (Dudgeon et al. 2006). Therefore, understanding the impacts of multiple stressors on changes in freshwater fish habitats is a current hotspot in ecosystem conservation and biodiversity research.

Hydropower dam construction is a prominent anthropogenic stressor that exerts profound and severe impacts on fish populations and river ecosystems (Ziv et al. 2012; Xie 2017). While providing clean energy and leveraging the comprehensive social and economic effects of water resources management, dam construction affects the natural morphology of rivers (Bice et al. 2014; Anderson et al. 2018), influences their hydrological rhythms (Deng et al. 2006; Cheng et al. 2015), blocks fish migratory corridors (Cao 2011), and shrinks running water habitats (Cheng et al. 2015; Timpe and Kaplan 2017), resulting in a significant decline in fish stocks (Gao et al. 2013). The upper Yangtze River basin (UYRB), a global biodiversity hotspot, has experienced extensive hydropower dam construction over the past two decades, severely impacting native fish habitats and biodiversity (Lin et al. 2019; Sabo et al. 2017; Zheng et al. 2024). Coupled with the impacts of climate change, the future alteration of fish habitat in the UYRB remains unknown and few researchers have addressed this issue (Zhang et al. 2019; Sun et al. 2020). Therefore, it is essential to explore the changes and driving mechanisms of fish habitats under complex changing conditions at a basin scale, which will furnish scientific reference to better coordinate and optimize the relationship between clean energy development and ecological conservation.

Over the past few decades, species distribution models (SDMs) have emerged as a crucial method for modeling species distributions under changing environments and climates (Booth et al. 2014; Elith and Leathwick 2009). By predicting the presence probability of species through time and space, biologists and managers can use SDMs to predict biological invasions, identify critical habitats, prioritize the locations of protected areas, and appropriately translocate endangered species (Guisan et al. 2013). Of these, the maximum entropy model (Maxent; Phillips, Anderson, and Schapire 2006) is characterized by its predictive power as the best performer in many contexts and has been widely used (Pearson et al. 2007; Morales, Fernández, and Baca‐González 2017). Currently, SDMs have been utilized in predicting and evaluating the effects of climate change and human activities (e.g., land use and cover change) on the distribution of freshwater fishes (Maloney et al. 2013; Liu et al. 2019). However, the impact of hydropower dams on watershed‐scale species distribution is often overlooked (Sun et al. 2020). Given that the construction and operation of hydropower dam can negatively affect the hydrological connectivity and physical habitat quality of rivers, incorporating the impact of hydropower dams in the model would facilitate the quantitative evaluation of fish habitat changes. Additionally, coupling the impacts of hydropower dams and climate change can help quantitatively disentangle their respective contributions, while few studies have addressed these points.

In this study, we explore establishing SDMs for pre‐dam and post‐dam periods based on fish presence data of separated periods for the UYRB. Since the impact of hydropower dams is implicitly included in the fish presence data, the model can be used to analyze the impact of hydropower dams on fish habitat. Two endangered fish species endemic to the UYRB, *Coreius guichenoti*, a representative warm‐water fish distributed in the lower UYRB, and *Schizopygopsis malacanthus*, a representative cold‐water fish distributed in the upper UYRB, were taken as the target species. The established models were then used to predict the future habitat suitability (HS) of the two fishes under the combined impact of climate change and hydropower dams, and their impacts on habitat shifts were evaluated and quantified. This study aims to provide a method that can quantify the impact of hydropower stations and climate change on fish habitats at the basin scale and to clarify the spatiotemporal habitat variation patterns of representative cold‐water and warm‐water fish in changing environments. The research can serve as a reference methodology for further studies on the species range and diversity under the impact of hydropower development and climate change, thereby providing fundamental support for effectively balancing hydropower development, water resource management, and ecological conservation.

## 2 Materials and Methods

### 2.1 Study Area and Target Species

**FIGURE 1:**
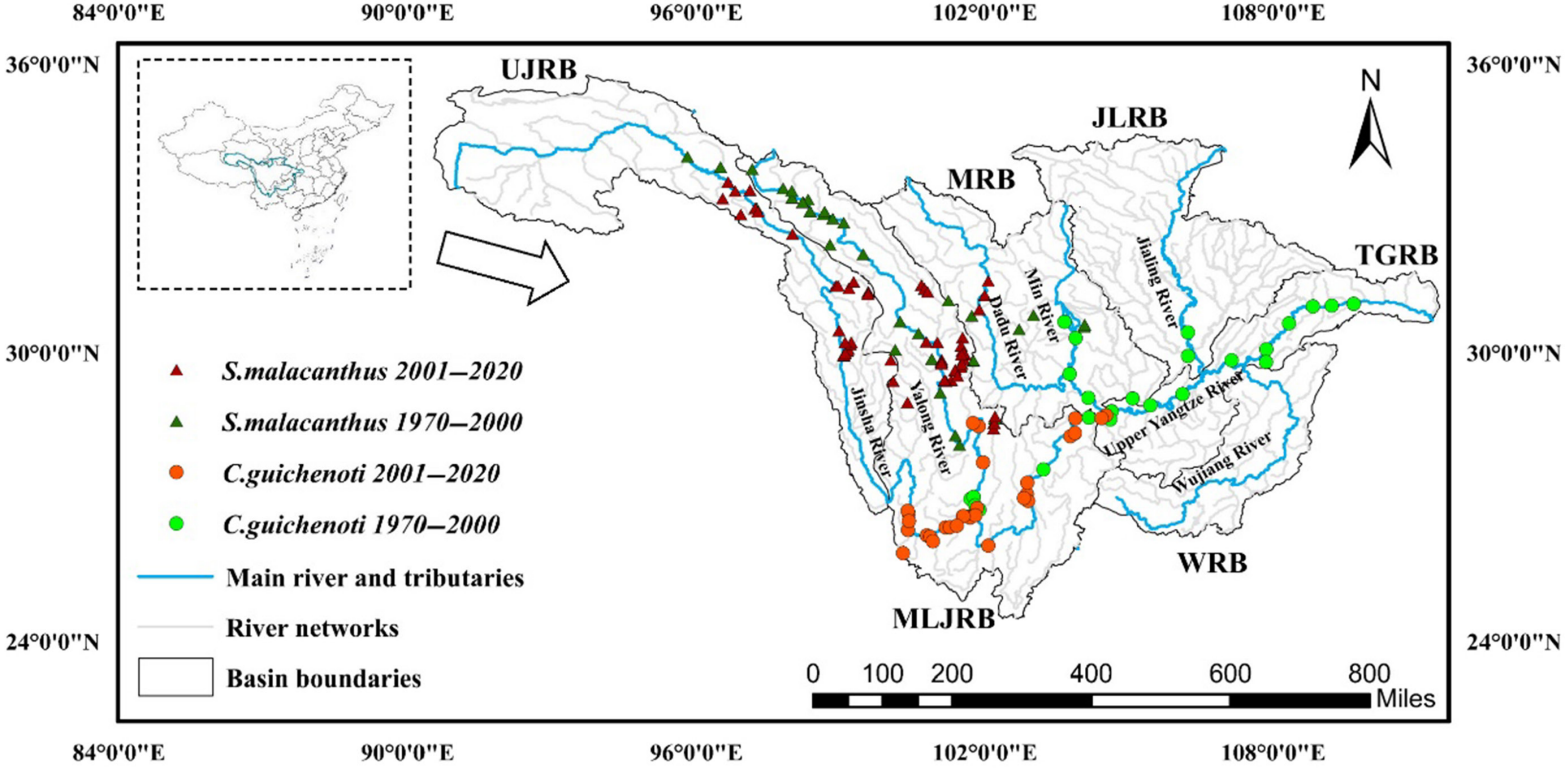
The location of the study area and the presence of *Coreius guichenoti* and *Schizopygopsis malacanthus* in the periods of 1970–2000 and 2001–2020.

The UYRB (97°37′–110°18′ E, 21°13′–34°33′ N), spanning from Tanggula Mountains on the Qinghai‐Tibet Plateau to Yichang City where the Three Gorges Dam is located, encompasses a basin area of approximately 1 million km^2^ and a total river length of 4511 km (Figure 1). The topography of the UYRB is highly undulating, with a relative altitude difference of 6790 m. The average annual temperature of the UYRB is approximately 12.5°C, with the average temperature of the coldest month being approximately 5°C or less. The annual precipitation ranges from 723 to 1134 mm, with the spatial distribution of precipitation showing a pattern of less in the west and more in the east, and the distribution of precipitation is not uniform and is mostly concentrated from April to October. The UYRB is divided into five important subbasins (Figure 1): the Upper Jinsha River basin (UJRB), the Middle and Lower Jinsha River basin (MLJRB), the Min River basin (MRB), the Jialing River basin (JLRB), the Wu River basin (WRB), and the Three Gorges Reservoir basin (TGRB).

Due to the varying gradients of climate, topography, and geomorphology from the headwaters to downstream areas, the UYRB is home to numerous endemic fish species, totaling 126, which represent 44.1% of the total fish species in the UYRB (Lin et al. 2019). Geographically, the upper reaches of the UYRB near the Tibetan Plateau are dominated by cold‐water fishes, and the middle and lower reaches are dominated by warm‐water fishes. To make the study more representative and convincing, *C. guichenoti* and *S. malacanthus*, two typical warm‐water and cold‐water fishes endemic to the UYRB, were selected as the target species of this study.

*Coreius guichenoti* belongs to the Cypriniforms order and Gobioninae family. It is an important economic fish endemic to the UYRB (Liu, Wu, and Wang 1990). It is a typical river migratory warm‐water fish that prefers flowing water and spawns drifting eggs, and the mature parents migrate upstream to the middle and lower reaches of the Jinsha River to complete reproduction. In recent years, due to the dam construction, the resources of *C. guichenoti* have declined drastically, and it was listed as critically endangered in 2016 (Jiang et al. 2016), and their conservation and restoration are extremely urgent (Chen and Zhang 2011).

*Schizopygopsis malacanthus* belongs to the Cypriniforms order and Schizothoracinae famliy, which is also an economically important fish (Ding 1994; He and Chen 2007). It is a typical cold‐water fish of the plateau (Yang et al. 2022) and is mainly distributed in the wide valley sections and higher elevation rivers of the upper Jinsha and Yalong Rivers (Le 2000). However, with the development of watersheds and the massive construction of barrages, dams, and reservoirs, the survival and reproduction of *S. malacanthus* are greatly affected (Li et al. 2022a, 2022b). *Schizopygopsis malacanthus* is affected by factors such as climate change, habitat destruction, and human activities, and its wild population size has declined dramatically which needs urgent protection and restoration (Jiang et al. 2016).

### 2.2 Species Presence Data Collection

The UYRB stands as a focal point for hydropower development in China, with 417 large‐ and medium‐sized hydropower dams planned to be built, of which a total of 127 cascade hydropower dams have been built for the main river and its large tributaries (Lin et al. 2019). Since only a few small dams were constructed before 2000 and most of the hydropower dams, especially huge dams, were commenced after 2000, we collected fish presence data during two distinct periods: the pre‐dam period (1970–2000) and the post‐dam period (2001–2020). The information on the impacts of dams on the species distribution is contained in the spatial distribution difference of the presence data of the two periods as there is no systematic bias in the sampling done in the two periods (Figure 1).

Species presence data for *C. guichenoti* and *S. malacanthus* were obtained from three main sources: (1) literature surveys; (2) GBIF (Global Biodiversity Information Facility, https://www.gbif.org/); and (3) fishing survey data from CAS (The Chinese Academy of Sciences). All records were checked for synonyms and naming errors, and the correctness of the spatial location of the records was checked before modeling (da Mata et al. 2017). To exclude the effects of spatial autocorrelation, presence data records were thinned (Boria et al. 2014; Aiello‐Lammens et al. 2015), and only one record was retained for a grid with a spatial resolution of 30 arcsec (Steen et al. 2021). After processing, a total of 59 records were retained for *C. guichenoti*, 29 for the pre‐dam period and 30 for the post‐dam period; a total of 92 records were retained for *S. malacanthus*, 35 for the pre‐dam period and 57 for the post‐dam period (Figure 1, Appendix S1). The occurrence points of the two periods have a good and similar marginal effect on environmental variables (Appendix S2), the models are believed to perform better (Tessarolo et al. 2021).

### 2.3 Predictor Variable Selection

The selection of predictor variables plays a pivotal role in accurately quantifying realized ecological niches, which is essential for ensuring the robustness and reliability of SDMs across different times and locations (Townsend Peterson, Papeş, and Eaton 2007). In this study, we fully considered the impact of environmental factors (Zhang et al. 2020) and selected 27 environmental variables, including 19 bioclimate variables, 4 human activity variables, and 4 hydro‐morphology variables, as predictors (Table 1). Bioclimate variables that have been commonly used in the field of ecological niche modeling (Hijmans et al. 2005) were obtained from the WorldClim database (http://www.worldclim.org/). These are a series of biologically significant temperature and rainfall characteristic that consist of 11 temperature‐related and 8 precipitation‐related variables. The bioclimate variables for the pre‐dam period (1970–2000) were directly downloaded from Worldclim, and the variables for the post‐dam period were calculated from the monthly mean maximum temperature, monthly mean minimum temperature, and monthly precipitation from 2001 to 2020 based on the biovars function in the R language “dismo” package. These data were downscaled from CRU‐TS‐4.06 (Harris et al. 2020) by Climate Research at the University of East England and bias‐corrected using WorldClim 2.1 (Fick and Hijmans 2017). The source of 4 human activity variables and 4 hydro‐morphology variables can be found in Appendix S3.

**TABLE 1:** Preselected predictor variable descriptions and sources.

Attributes	Variables	Description
Bioclimate	BIO1	Annual mean temperature
	BIO2	Mean diurnal range
	BIO3	Isothermality
	BIO4	Temperature seasonality
	BIO5	Max temperature of warmest month
	BIO6	Min temperature of coldest month
	BIO7	Temperature annual range
	BIO8	Mean temperature of wettest quarter
	BIO9	Mean temperature of driest quarter
	BIO10	Mean temperature of warmest quarter
	BIO11	Mean temperature of coldest quarter
	BIO12	Annual precipitation
	BIO13	Precipitation of wettest month
	BIO14	Precipitation of driest month
	BIO15	Precipitation seasonality
	BIO16	Precipitation of wettest quarter
	BIO17	Precipitation of driest quarter
	BIO18	Precipitation of warmest quarter
	BIO19	Precipitation of coldest quarter
Human activity	CLA	Crop land area
	DPI	Development pressure index
	HIP	The human impervious area percentage
	POPC	Population count
Hydro‐morphology	DEM	Digital elevation model
	FHF	Flood hazard frequency
	RO	River order
	WTA	Water area

**FIGURE 2:**
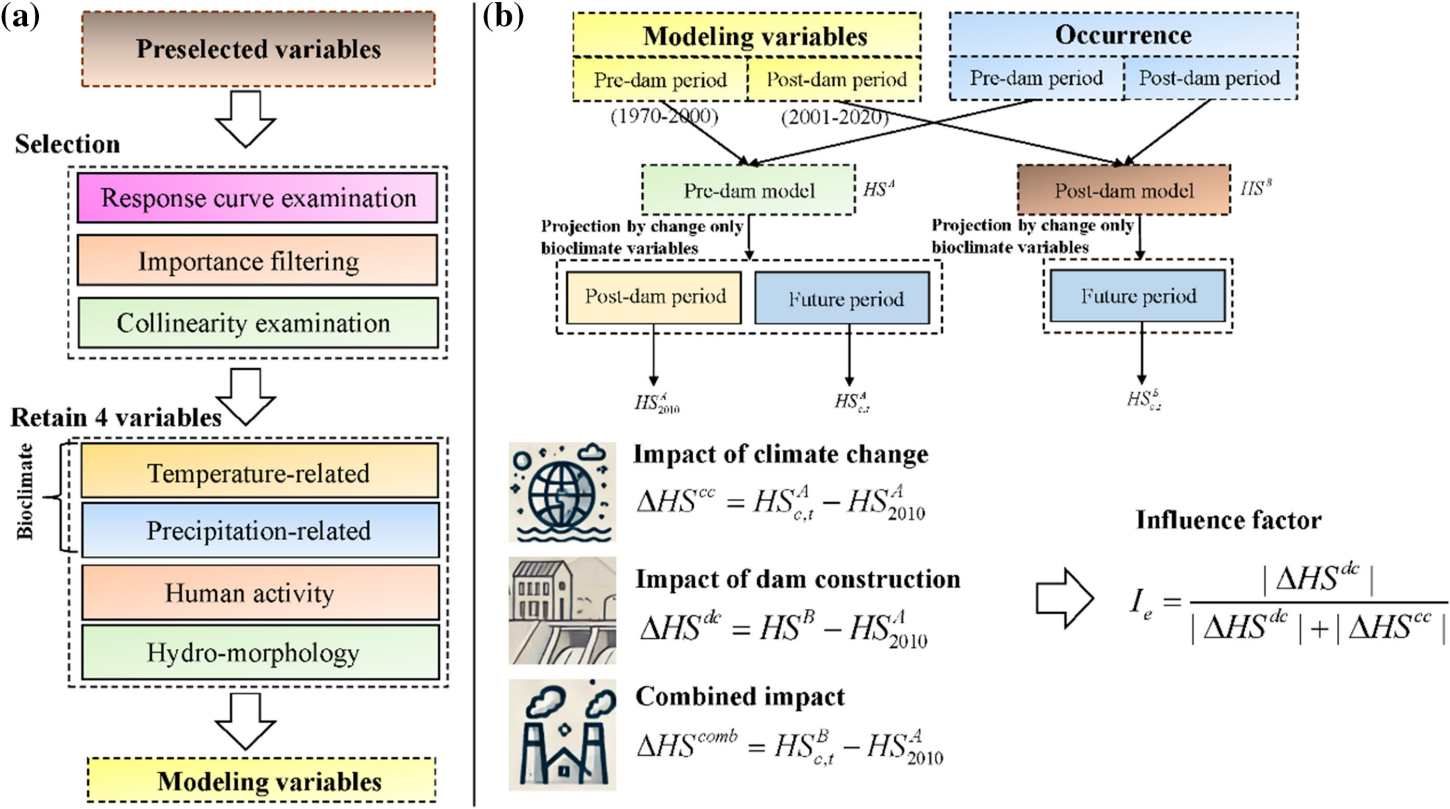
Modeling variables selection process (a) and impact analysis process (b).

We select predictor variables in three steps (Figure 2a). First, the resulting response curves from the premodeling of the pre‐dam and post‐dam periods are examined to remove variables with non‐monomodal or non‐monotonically varying response curves and select variables with similar response curves for the two time periods. Then, a knife‐cut method (Alkhalifah et al. 2023) was adopted to synthesize the contribution and replacement importance of the variables ensuring that at least one representative variable from each category—temperature‐related bioclimate variables, precipitation‐related bioclimate variables, human activity variables, and hydro‐morphology variables—was retained in the model. Finally, to prevent overfitting and mitigate the influence of collinearity, the variance inflation variable (VIF) was calculated for the selected variables to retain only those variables with a VIF < 10 (De Marco and Nóbrega 2018). Ultimately, the mean temperature of the driest quarter (BIO9), annual precipitation (BIO12), development pressure index (DPI), and river order (RO) were selected for the *C. guichenoti*; the mean temperature of the driest quarter (BIO9), precipitation seasonality (BIO15), cropland area (CLA), and digital elevation model (DEM) were retained for the *S. malacanthus*. These variables were then used in the Maxent modeling analysis.

### 2.4 Maxent Modeling

To build the Maxent model, we used open‐source Maxent software (version 3.4.3, November 2020; http://biodiversityinformatics.amnh.org/open_source/maxent). The framework incorporates some of the most recent advances in the field of SDMs by using background points instead of pseudo‐absence points (different from pseudo‐absence points, but both are used in place of true absence points; Dong et al. 2024). Background points can effectively reduce the inherent sampling bias in presence records, thereby significantly improving the predictive performance of models (Kramer‐Schadt et al. 2013; Varela et al. 2014; Castellanos et al. 2019). We constructed the Maxent model following the standard procedure (Dong et al. 2024): First, 10,000 background points were generated. Second, the presence dataset was randomly divided into a training set (70%) and a test set (30%). Then, the area under the curve (AUC) of the receiver operating characteristic (ROC) were chosen to evaluate the performance of the model. Lastly, response curves were used to show the marginal effects of all variables on *C. guichenoti* and *S. malacanthus* over 1970–2000 and 2001–2020. This process was then repeated 10 times to reduce the bias in splitting the dataset caused by the variability of individual algorithms and to increase the rigor of the results. In this case, the AUC ranged from 0.5 to 1.0, with values > 0.9 indicating good model performance (Mulieri and Patitucci 2019).

### 2.5 Model Prediction

Using the Maxent model and the selected predictors, models for the pre‐dam and post‐dam periods were established for both *C. guichenoti* and *S. malacanthus*. Based on these models, projections are made on future bioclimate data for 2050 (average of 2041–2060) and 2090 (average of 2081–2100) by withholding variables other than climate constants. It is reasonable for RO and DEM to be held constant in the model as hydro‐morphology variables. In addition, since there are no future DPI or CLA datasets available, we kept these variables constant in future projections. Indeed, the use of static non‐climatic variables in predicting climate change impacts is widely accepted (Peterson et al. 2002). It is believed that only changing the future bioclimate variables in the prediction is essential for evaluating the impacts of future climate change. Previous studies have also shown that models that include both static and dynamic variables perform better than those that mask or exclude static variables (Iverson and Prasad 1998; Stanton et al. 2012). To reduce the uncertainty in the projection of future scenarios, we selected meteorological products from two widely used global circulation models (GCMs): MIROC‐ES2L and CNRM‐ESM2‐1 (Appendix S4). Two shared economic pathway (SSP) climate scenarios, SSP1‐2.6 and SSP5‐8.5, which simulate optimistic scenarios for sustainable development and pessimistic scenarios driven by fossil fuel development, respectively (Zhou et al. 2021), were considered to project future fish habitat under the different levels of climate change impacts. As a result, model predictions in eight scenarios that combine two future periods, two climate models and two climate scenarios under the impacts of climate change without and with the impact of dams for each fish species were used for impact analysis.

### 2.6 Impact Analysis

The flowchart in this section refers to Figure 2b. The distribution of HS for a given period under a given climate scenario is expressed as ${\mathrm{HS}}_{c,t}^{p}$ where *c* represents the climate scenario with two SSPs; *p* represents the model for prediction, with A denoting the pre‐dam model (using occurrence datas and climate variables in 1970–2000) and B denoting post‐dam model (using occurrence datas and climate variables in 2001–2020), and *t* represents the corresponding period that model *p* projected to the climate variable. Here, we used the pre‐dam model projection for the post‐dam period that only changed the climate variables from 2001 to 2020 (marked as 2010) as the basal model. The habitat changes under the separated and combined impacts of dam construction climate change were used for impact analysis, and they are calculated as follows:

(1) $$\Delta {\mathrm{HS}}^{\mathrm{cc}}={\mathrm{HS}}_{c,t}^{A}-{\mathrm{HS}}_{2010}^{A}$$

(2) $$\Delta {\mathrm{HS}}^{\mathrm{dc}}={\mathrm{HS}}^{B}-{\mathrm{HS}}_{2010}^{A}$$

(3) $$\Delta {\mathrm{HS}}^{\text{comb}}={\mathrm{HS}}_{c,t}^{B}-{\mathrm{HS}}_{2010}^{A}$$

where ${\mathrm{HS}}_{2010}^{A}$ denote the basal model projection; the impact of climate change on HS $\Delta {\mathrm{HS}}^{\mathrm{cc}}$ is measured as the difference between future projections with the pre‐dam model and the basal model projection; the impact of dam construction $\Delta {\mathrm{HS}}^{\mathrm{dc}}$ is measured as the difference between the post‐dam model results and the basal model; their combined impact is measured as the difference between future projections with post‐dam model and the basal model results.

In addition, to measure the extent of the impact of climate change or dam construction in a future scenario under the combined impact of climate change and dam construction, an impact factor $I_{e}$ is used, which is calculated as follows:

(4) $$I_{e}=\frac{|\Delta {\mathrm{HS}}^{\mathrm{dc}}|}{|\Delta {\mathrm{HS}}^{\mathrm{dc}}|+|\Delta {\mathrm{HS}}^{\mathrm{cc}}|}$$

where $I_{e}$ denotes the proportion of habitat changes caused by dam construction within the combined impacts of dam construction and climate change. A higher value of the $I_{e}$ signifies a more significant impact of the dam on habitat changes, whereas a lower value indicates less impact of the dam but more by climate change. The predominant impacts of climate change or dam construction were determined by classifying the impact factor into different levels. The degree of the predominant impacts of hydropower development and climate change are each divided into two levels, which are shown in Table 2.

**TABLE 2:** Impact factor $I_{e}$.

Value	Evaluation results	Level
0% ≤ $I_{e}$ < 20%	Almost exclusively influenced by climate change	C2
20% ≤ $I_{e}$ < 40%	Mainly influenced by climate change	C1
60% ≤ $I_{e}$ < 80%	Mainly influenced by the dam construction	D1
80% ≤ $I_{e}$ < 100%	Almost exclusively influenced by the dam construction	D2

## 3 Results

### 3.1 Model Performance and Variable Contribution

**FIGURE 3:**
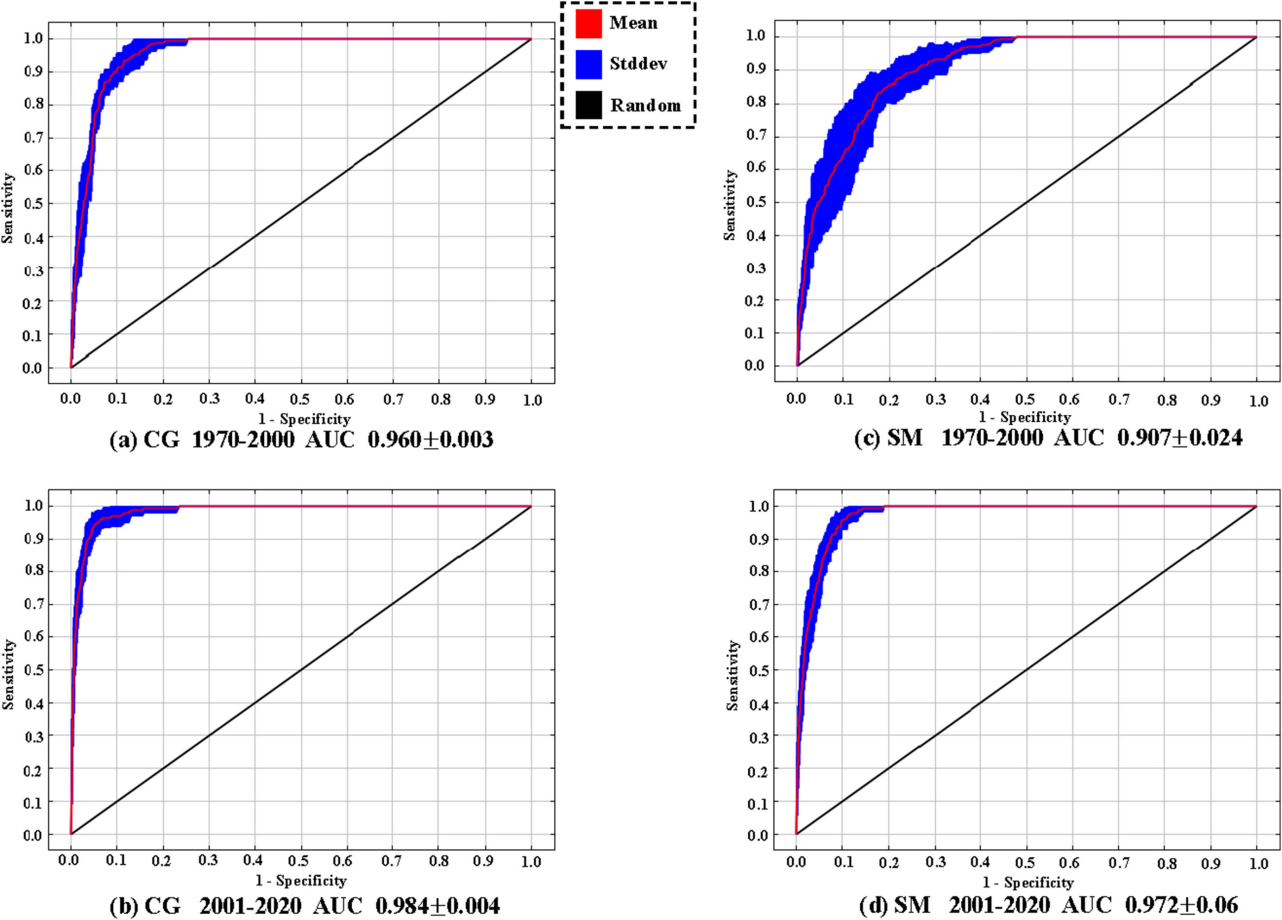
AUC of the Maxent model for *Coreius guichenoti* (CG) and *Schizopygopsis malacanthus* (SM) for the pre‐dam (1970–2000) and post‐dam (2001–2020) periods.

The results showed that the pre‐dam and post‐dam Maxent models performed well both for *C. guichenoti* and *S. malacanthus*, with an average AUC above 0.9 for all 10 runs of each model (Figure 3). The AUC is significantly greater than the purely speculative threshold of 0.5, implying that our model is likely to be accurate and reliable for predicting the current and future spatial distributions of the species in question.

The results of the variable importance tested by the knife‐cut method are shown in Appendix S5. For the selected four variables, BIO9 is the main bioclimatevariable that contributed to the occurrence of *C. guichenoti* both in the pre‐dam period (variable importance: 32.5%) and post‐dam period (47.6%); RO is the main non‐climatic variable that impacts *C. guichenoti* with the variable importance was 41.4% and 36.7%, respectively, in the pre‐dam and post‐dam periods. BIO15 is the crucial bioclimate variable, and the CLA is the main non‐climatic variable that dominates the species distribution of *S. malacanthus*. The importance of BIO5 was 45.1% and 27.2%, and the importance of CLA was 28.3% and 33.4%, respectively, for the pre‐dam and post‐dam periods. The response curves of all the selected variables can be seen in Appendix S2.

### 3.2 Historical Habitat Changes Under Dam Construction

**FIGURE 4:**
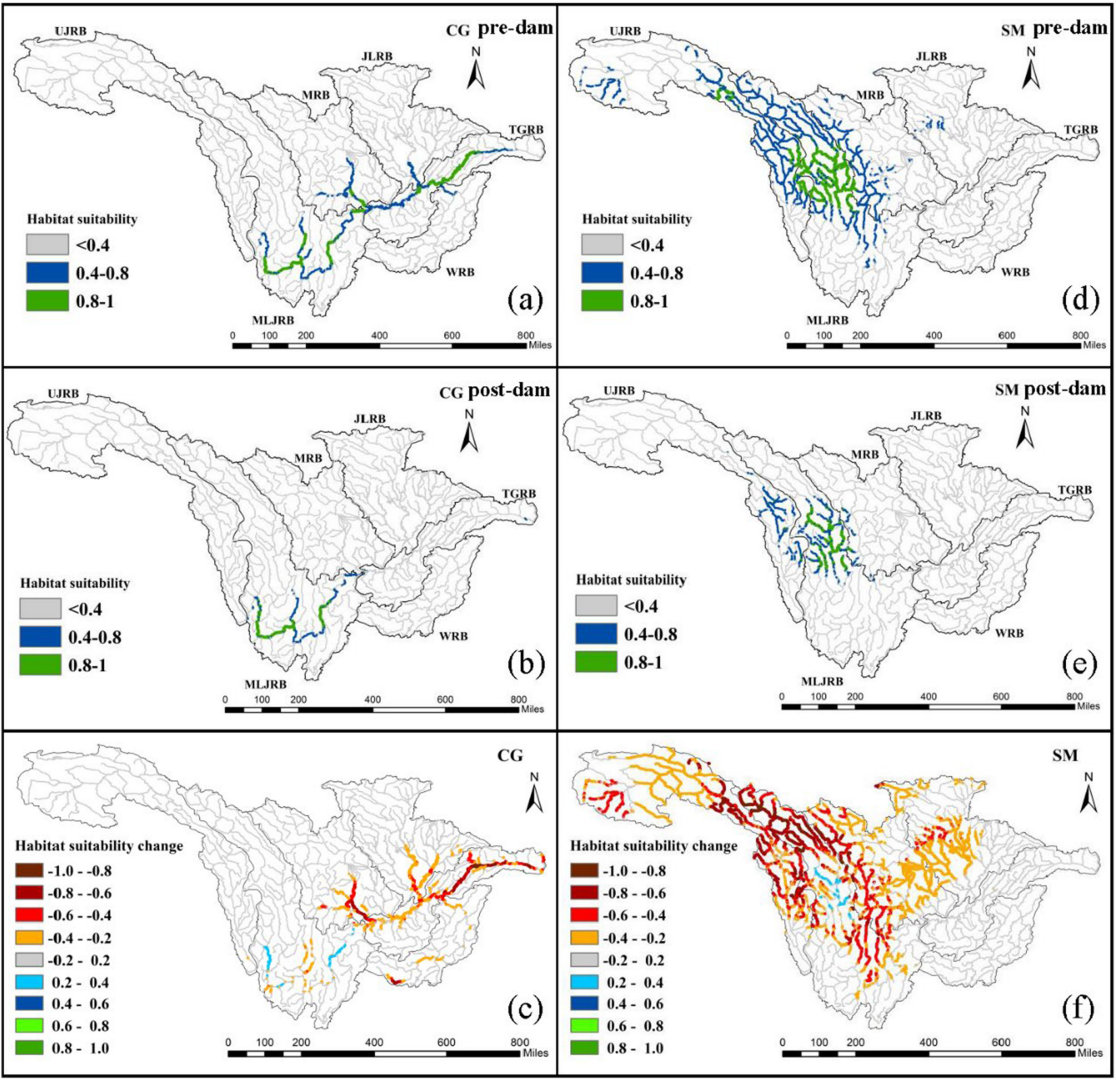
HS distribution of *Coreius guichenoti* (CG) and *Schizopygopsis malacanthus* (SM) for the pre‐dam (1970–2000) and post‐dam (2001–2020) periods (a, b, d, e) and HS changes under the impact of dam construction (c, f).

Comparisons of model results in pre‐dam and post‐dam periods showed that the HS of both *C. guichenoti* and *S. malacanthus* experienced significant reductions after 2000 (Figure 4). For *C. guichenoti*, the high‐suitability area (HSA, HS ≥ 0.8) remained only in the Jinsha River and Yalong River after 2000, but it disappeared in the lower UYRB, especially in the main reach of the Yangtze River. The difference in simulation results caused by dam construction showed that the HS in the mainstream of the Three Gorges Reservoir area and the lower Min River decreased by more than 0.6, while it was slightly increased in some sections of the middle and lower reaches of the Jinsha River (Figure 4c). The suitable habitats of *S. malacanthus* were widely distributed in the upper reaches of the Jinsha River and the middle and upper reaches of the Yalong River before 2000, but it shrunk towards the centers of their distribution range under the impact of dam construction (Figure 4e). After 2000, only the middle reaches of the Yalong River retained HSA. The negative impacts of dam construction on *S. malacanthus* are widespread, and the suitability around the HSA was significantly decreased by more than 0.4 (Figure 4f).

**FIGURE 5:**
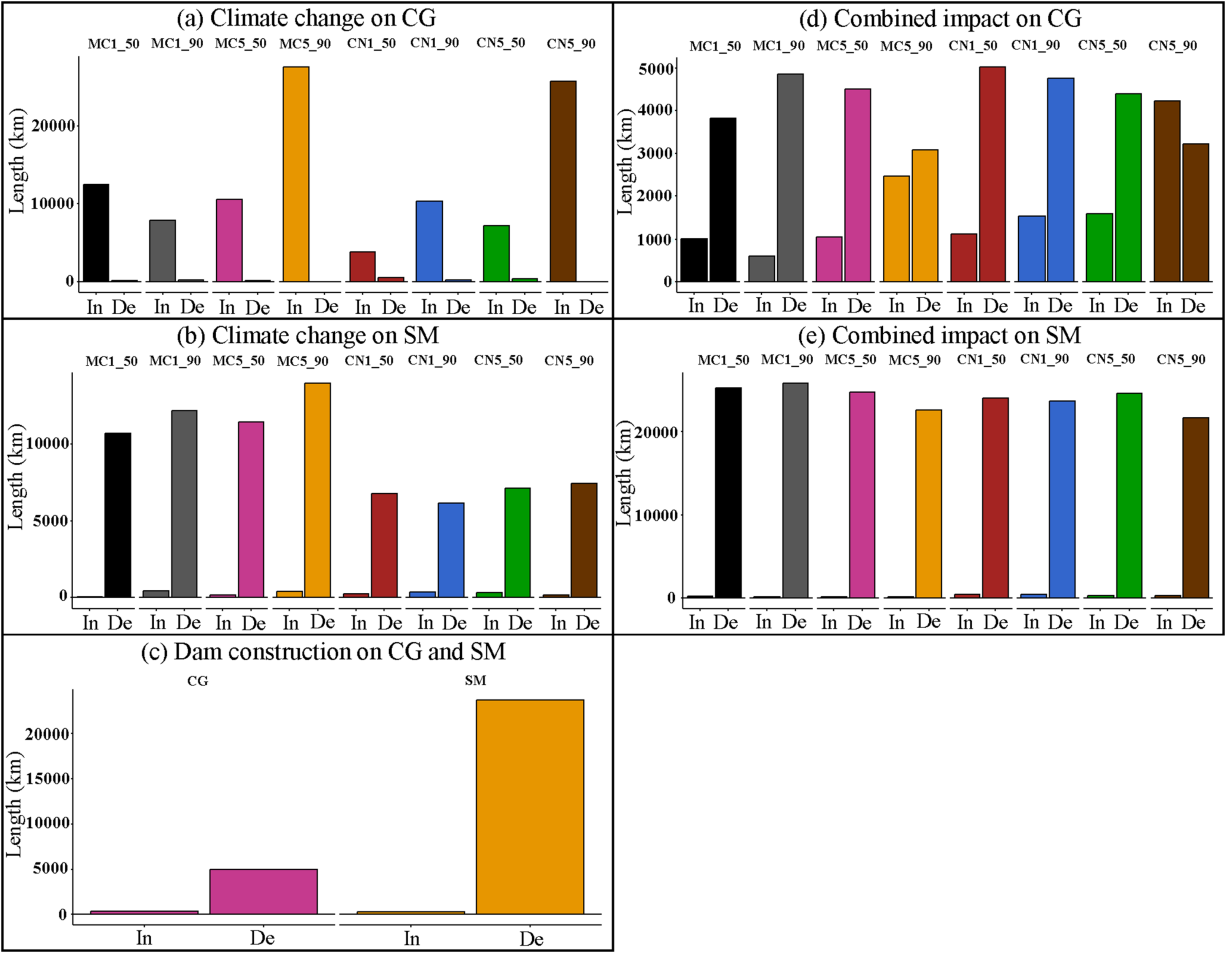
River length of high‐suitability area (HSA) increased (In) and decreased (De) by climate change, dam construction, and their combined impacts (50 denotes 2050; 90 denotes 2090; CN1 denotes CNRM‐ESM2‐1 under SSP1‐2.6; CN5 denotes CNRM‐ESM2‐1 under SSP5‐8.5; MC1 denotes MIROC‐ES2L under SSP1‐2.6; MC5 denotes MIROC‐ES2L under SSP5‐8.5).

The river length of decreased and increased HS under the separated and combined impact of dam construction and climate change was quantified (Figure 5). The river lengths where the fish HS is reduced due to dam construction were 5009.0 and 23,745.0 km, respectively, for the *C. guichenoti* and *S. malacanthus* (Figure 5c,f). The HSA of the *C. guichenoti* decreased by 56.3% from 1279.9 to 559.7 km between pre‐dam and post‐dam periods (Figure 4a,b). The river length of HSA for *S. malacanthus* decreased from 2485.34 to 819.81 km, with a reduction of 67.0% between pre‐dam and post‐dam periods (Figure 5d,e). The historical habitat changes of these two representative cold‐water and warm‐water fish species highlight the profound adverse impacts of dam construction on the endemic fishes of the Jinsha River.

### 3.3 Future Habitat Shifts Under Climate Change

**FIGURE 6:**
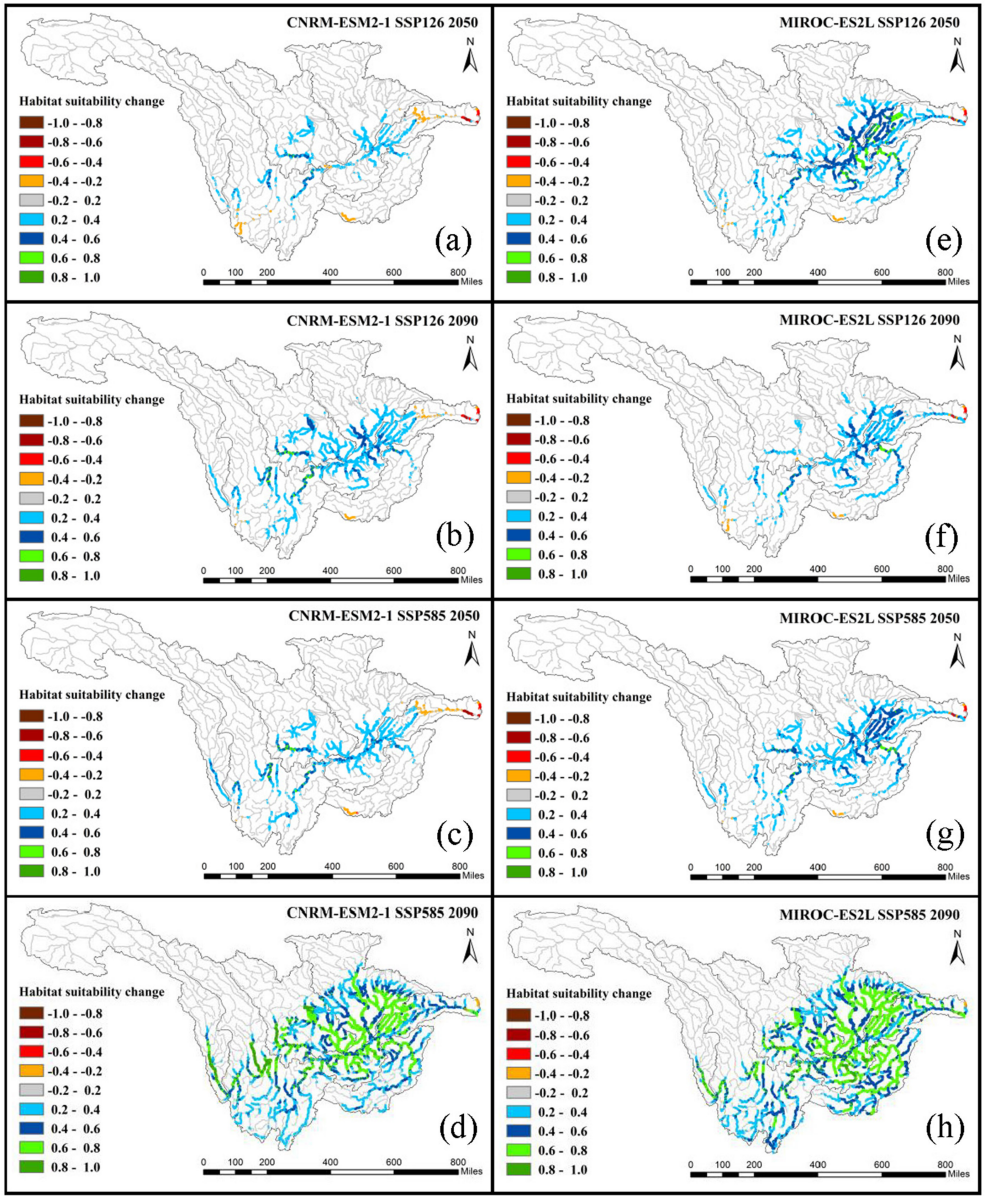
HS changes of *Coreius guichenoti* under the impacts of climate change.

**FIGURE 7:**
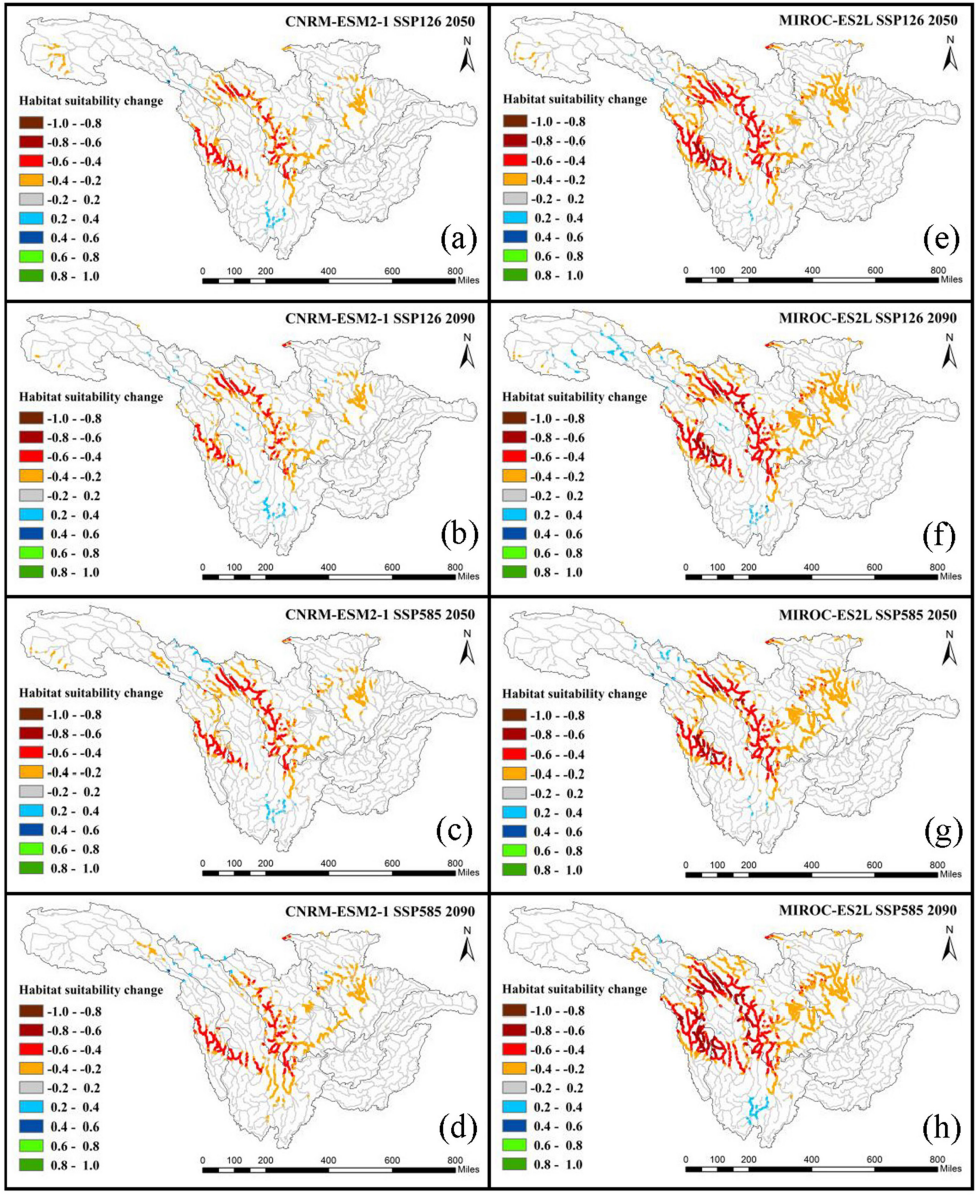
HS changes of *Schizopygopsis malacanthus* under the impacts of climate change.

The distributions of habitat change in the 2050s and 2090s under the SSP126 and SSP585 scenarios are shown in Figure 6 (*C. guichenoti*) and Figure 7 (*S. malacanthus*). Across the whole basin, all climate change scenarios exert positive impacts on *C. guichenoti*, leading to a noticeable increase in HS for originally suitable habitat areas and a habitat expansion to upstream rivers and tributaries. Under different climate scenarios, although the extent and degree of the increase in HS varied, the overall trends are essentially consistent. The most significant increase in HS of *C. guichenoti* occurred in the 2090s under the SSP585 scenario in both climate models. In contrast, climate change will induce a negative impact on the *S. malacanthus* with the HS decreased by more than 0.4 in most areas. Like the impacts of dam construction (Figure 3f), the HS significantly decreased at the edges of HSA centers, indicating that climate change also leads to the contraction of HSA. Although MIROC‐ES2L resulted in a greater decrease in HS, both climate models predicted a similar decreasing direction of suitable habitat. These different habitat shift patterns between *C. guichenoti* and *S. malacanthus* indicate that climate change would induce opposite impacts on cold‐water and warm‐water fishes in the UYRB.

### 3.4 Future Habitat Shifts Under Combined Impacts

**FIGURE 8:**
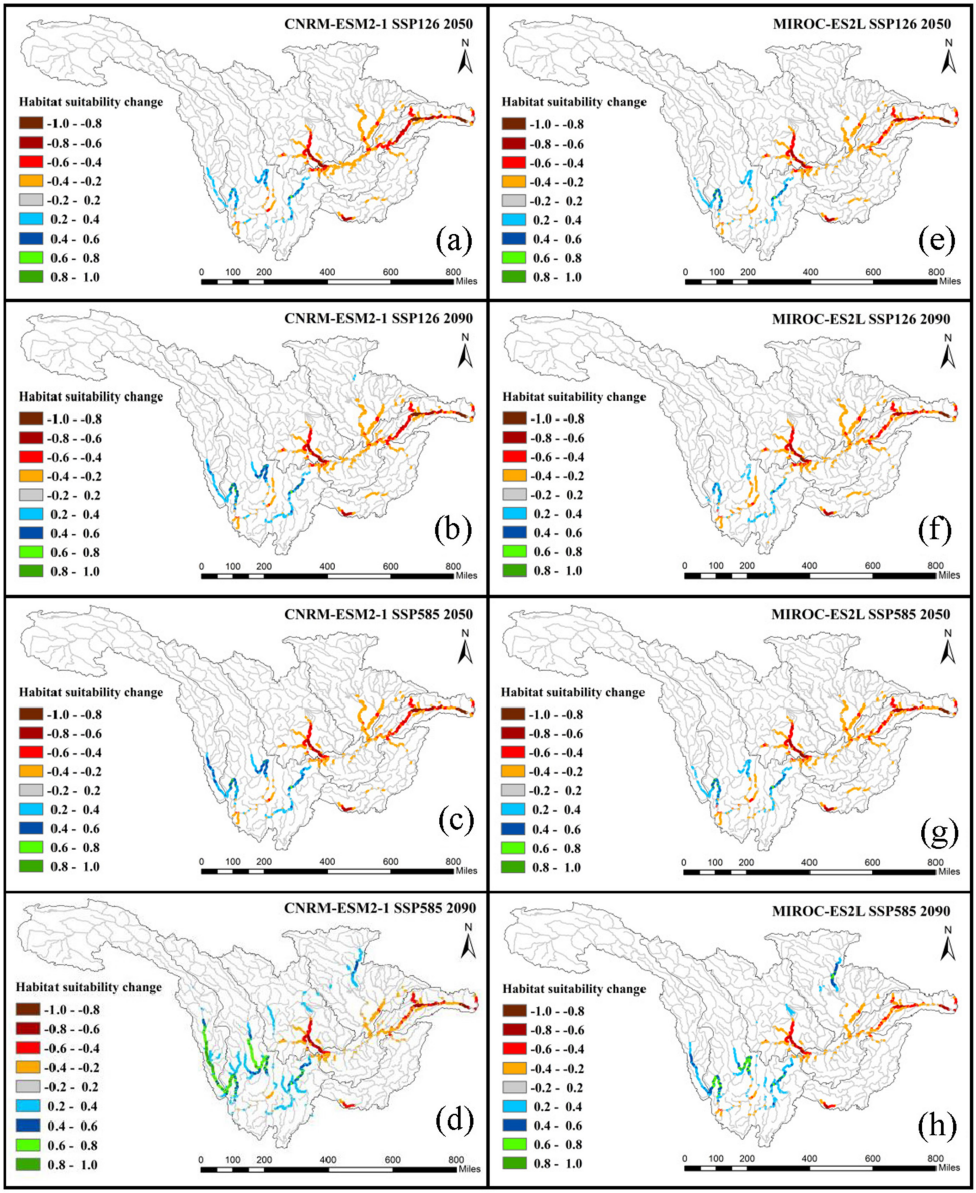
HS changes of *Coreius guichenoti* under the combined impact of all variables.

**FIGURE 9:**
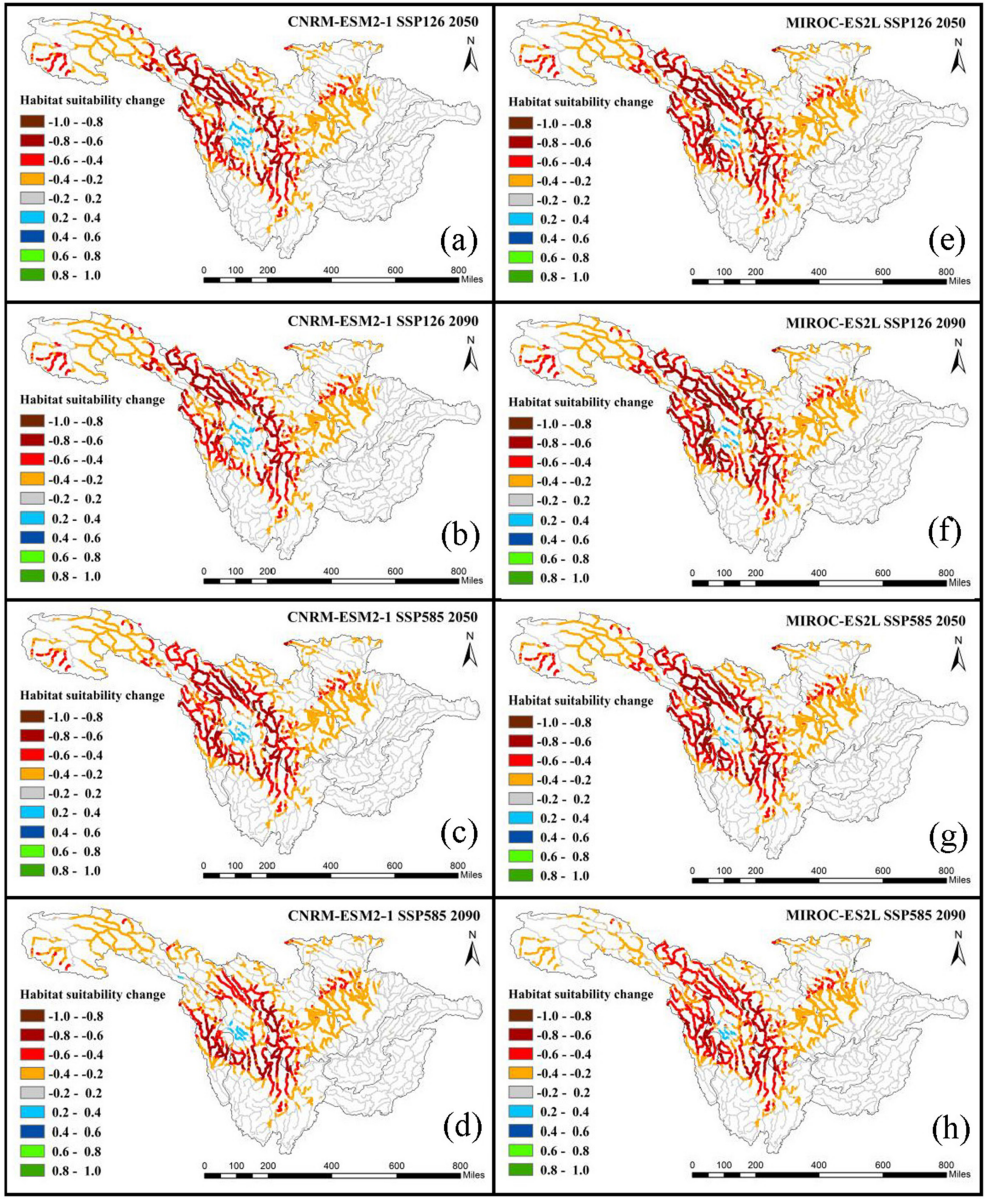
HS changes of *Schizopygopsis malacanthus* under the combined impact of all the variables.

The spatial distribution of HS changes in the 2050s and 2090s under the combined impacts of dam construction and climate change under SSP126 and SSP585 scenarios on *C. guichenoti* and *S. malacanthus* are shown in Figures 8 and 9, respectively. For the *C. guichenoti*, the distribution pattern of decreased habitat areas impacted by the combined variables in the lower reaches of the UYRB is almost identical to that only impacted by dam construction (Figure 4c). This indicates that the positive impacts of climate change (Figure 5) have not mitigated the negative impacts of dam construction in that reaches. However, the positive impacts of climate change under the combined impacts are apparent in the middle and lower reaches of the Jinsha River, with areas that have experienced a decrease in HS due to the dam construction seeing an increase in HS under the impact of climate change. Suitable habitats tend to further expand upstream of the Jinsha and Yalong Rivers and their tributaries, especially under the high SSP scenario by 2090.

The fish habitat shifts of *S. malacanthus* under the combined impacts of climate change and dam construction (Figure 8) show that the decreasing patterns in HS are very similar to the scenario only impacted by dam construction (Figure 3f). The negative impacts induced by climate change may have led to a further decrease in the extent and range of HS. The suitability in the HSA center would slightly increase under the combined impacts, but as climate change intensifies and pushes forward to the far future (SSP585 in the 2090s, Figure 9d,h), this positive impact will gradually decrease.

### 3.5 Dominant Impact Factor Analysis

**FIGURE 10:**
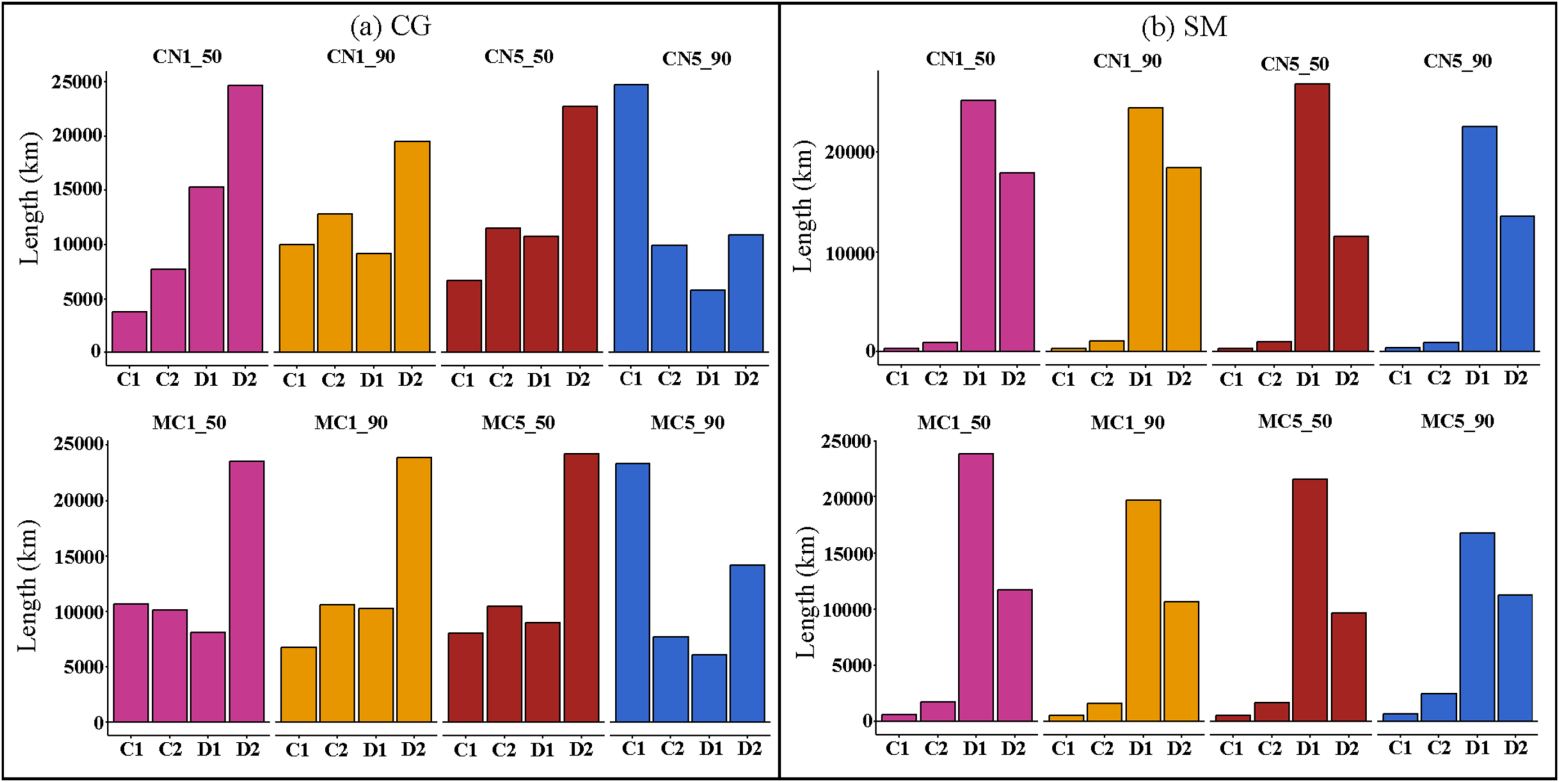
Comparisons of river lengths that are predominately influenced by climate change and dam construction under the combined impacts for *Coreius guichenoti* (CG) and *Schizopygopsis malacanthus* (SM) (50 denotes 2050; 90 denotes 2090; CN1 denotes CNRM‐ESM2‐1 under SSP1‐2.6; CN5 denotes CNRM‐ESM2‐1 under SSP5‐8.5; MC1 denotes MIROC‐ES2L under SSP1‐2.6; MC5 denotes MIROC‐ES2L under SSP5‐8.5; the definition of C1, C2, D1, and D2 can be found in Table 2).

After calculating the impact factor $I_{e}$, the river lengths of the exclusively influenced (D2 or C2) and mainly influenced (D1 or C1) areas by dam construction or climate change were summarized and compared in Figure 10. It was shown that, for the *C. guichenoti*, the river reaches exclusively influenced by dam construction (D1) were significantly higher than that dominantly impacted by climate change except in 2090 under SSP585 scenarios in the two GCMs. Under the combined impacts, the predominantly influenced area by dam construction outweighed that of climate change with the former averaging 1.97 times greater for *C. guichenoti* (Appendix S6). Both the exclusively influenced (D2) and mainly influenced (D1) areas by dam construction were vastly higher than those impacted by climate change for *S. malacanthus* (Appendix S7), with the river lengths that predominately influenced by dam construction averaging a minimum of 8.81 times (MIROC‐ES2L SSP585 2090) and a maximum of 32.62 times greater (CNRM‐ESM2‐1 SSP126 2050) than those by climate change. This indicates that the negative impacts of dam construction on fish habitats will still dominate in future climate change periods.

## 4 Discussion

### 4.1 Impacts of Dam Construction on Fish Habitat

In the past two decades, the construction of cascade hydropower reservoirs in the UYRB, including the Three Gorges Reservoir (TGR), has been the foremost factor in the significant reduction of fish resources and diversity, leading to the extinction of some species and making many fish species critically endangered (Dudgeon 2011). Dams affect fish in various ways, the most direct of which is altering the river's physical structure, hindering the ways for fish migration and dispersal. The change in physical structure also causes several functional impacts, such as disruptions to the natural flow regime for fish spawning, degradation and fragmentation of fish habitat, and invasion of non‐native fish to the reservoir (Chen et al. 2023). Reproduction is an important stage in the completion of the life cycle and the maintenance of population growth for fish. The *C. guichenoti* initiates spawning stimulated by suitable flowing water when the water temperature reaches above 18°C in April of each year. The most severe impact of dams on *C. guichenoti* is the loss of spawning grounds widely distributed in the middle and lower reaches of the Jinsha River and the Yalong River due to the cascade dam construction (Wang et al. 2020). This is mainly due to the loss of lotic habitats and the release of cold water from reservoirs in the summer, which significantly narrows the suitable temperature window for spawning *C. guichenoti*. Additionally, the nursery habitats of *C. guichenoti* have significantly decreased in the TGR and adjacent river reaches due to the reservoir's impoundment (Gao et al. 2010), which is consistent with our model results (Figure 3b). Research on the impact of dam construction on *S. malacanthus* is rare, possibly due to the greater difficulties in sampling and monitoring these benthic fish species on the plateau rivers. However, numerous studies have reported that the fish resources of Schizothoracinae fishes in the UYRB have been negatively affected by the cascade hydropower dams of the Jinsha River (Li, Li, and Li 2012; Lin et al. 2019).

Habitat models are important tools for assessing the impact of dams on fish habitats, and there is currently a wealth of research in this area (Yi et al. 2017). Such researches consider fish habitat preference for water depth, flow rate, substrate, etc., to simulate HS and assess the impact of hydropower station construction and operation on fish habitats based on physical habitat models (Zhang et al. 2018). However, since it requires an understanding of the physical background conditions of hydrology and hydraulics within the river channel in advance, it is difficult for such models to support basin‐scale simulations and analyses. Therefore, most related studies are focused on assessing the impact of hydropower station construction on the micro‐habitats of fish during a specific life stage in a particular river section. Our previous research coupled downscaled climatic models and multiscale hydrological and environmental models to study changes in river flow and water temperature under the impacts of climate change and hydropower development within the basin and evaluated the climate change and hydropower impact on spawning and juvenile fish habitat of *C. guichenoti*. However, this research only used the simulated results of the physical background at a specific river section for habitat assessment (Zhang et al. 2019). In the present study, our model has reasonably identified the negative impacts of hydropower dams on typical cold‐water and warm‐water fish and their spatial variation patterns on a basin scale. It remains challenging to assess the impact of hydropower dams on fish habitats by considering changes in the micro‐habitat conditions within the river channel from a whole basin perspective. Nonetheless, we still call for future research to undertake basin‐scale habitat simulations and assessments based on the hydrological and hydraulic conditions of river channels under the impact of hydropower development.

### 4.2 Impacts of Climate Change on Fish Habitat

Freshwater ecosystems could be those most threatened by the effect of future climate change (Millenium Ecosystem Assessment 2005). As ectothermic animals and key organisms in freshwater ecosystems, fish have been inevitably facing survival pressures due to changes in regional temperature and precipitation patterns caused by climate change which could force fish to migrate to find suitable areas in river systems. Observed and predicted trends in the distribution of freshwater fish have highlighted the fact that, in recent years, freshwater fish distributions have already been affected by contemporary climate change in ways consistent with anticipated responses under future climate change scenarios (Comte et al. 2013). Cold‐water fish species could be negatively affected by future climate changes, and the range of most cold‐water species could be reduced or shift to higher altitudes or latitudes (e.g., salmonids). In contrast, warm‐water species (e.g., Cyprinidae) could benefit from climate warming, and their habitat range would expand. Although our study did not consider a sufficient amount of cold‐water and warm‐water fish species for predictive analysis, the results are still consistent with the mainstream trends of climate change impacts, reflecting the future trend of habitat shifts for cold‐water and warm‐water fish in the UYRB. The warm‐water *C. guichenoti* has a wide suitable temperature range in its life history and the highest tolerance temperature for respiration and metabolism can reach 30°C (Duan et al. 2022). Our model has identified that the mean temperature of the driest quarter (BIO9) is the most important bioclimate variable affecting the habitat distribution of *C. guichenoti*. The dry season in the UYRB lasts from December to April of the following year (Chen et al. 2001), a period coinciding with late winter and early spring when temperatures are low. With the impact of climate change, warming winter would become more conducive to the survival of *C. guichenoti*, which would lead to a rapid expansion of its suitable habitat under future climate change, even in extreme climate change scenarios (RCP8.5) that fishes previously distributed in the lower reaches may extend their range to the upstream main streams or tributaries (Figure 5d,h). Conversely, the cold‐water fish *S. malacanthus*, a key species in the Tibetan Plateau, faces severe habitat reduction due to climate change. As identified by our model, the changes in the precipitation seasonality (BIO15) and warming winter temperatures (BIO9) in the dry season may be the main reasons for the significant reduction in the HS of *S. malacanthus*. It is worth noting that suitable habitats for *S. malacanthus* would not shift to higher altitudes or higher latitudes under the impact of climate change, but have instead continuously contracted towards the center. As climate change continues to intensify, habitat loss may put this species at risk of extinction. The richness of cold‐water and warm‐water fish in the UYRB are equally high. If these two kinds of fish evolve in completely different directions under future climate change, the overall species richness and diversity of the region will be greatly affected, and the balance and stability of the basin ecosystem will also be disrupted. Future research may need to focus on multiple species, as well as the interactions between species in the prediction model to explore the response of fish community to future climate change.

### 4.3 Implications and Limitations

There is a long history of research addressing the effect of large‐scale environmental change on freshwater fish habitat or range distribution, but most studies focus on the impacts of climate change (Lima et al. 2022; Morid, Shimatani, and Sato 2020). Although the impact of dams on aquatic life has received widespread attention globally, there are few studies on the large‐scale simulation and evaluation of the impact of hydroelectric development on biological habitats. This study uses the information on the impact of hydropower dams that is implicit in species occurrence data to conduct habitat assessments at the basin scale, providing a new solution for evaluations under the combined impacts of climate change and hydropower construction. Through our experimental simulation analysis, we found that the negative impacts of hydropower stations on cold‐water and warm‐water fish predominate in current and future periods. This requires us to pay more attention to fish habitat conservation and integrated management in the cascade development of hydropower dams. Notably, our study found that cold‐water fish in the upper reaches of the basin are more susceptible to the negative impacts of hydropower stations, and climate change would further deepen these negative impacts. Therefore, urgent measures are needed for the conservation and habitat restoration of plateau cold‐water fish species, including creating protected areas and implementing adaptive management strategies. Amidst the continuous cascade development in the upper Jinsha River, the future survival of these plateau cold‐water fish could be severely jeopardized if protective measures are not implemented.

We only used AUC to assess the model's performance. In fact, eco‐geographic and other data characteristics also influence the accuracy of SDM predictions (Tessarolo et al. 2021). The distributions of freshwater fishes are influenced by many factors operating at different scales. At a regional scale, freshwater fish distributions are influenced by hydro‐morphology and climatic factors. Local‐scale distributions could be influenced by abiotic (e.g., flow discharge and hydrodynamics) and biotic (e.g., species interactions and bait resources) (Chu, Mandrak, and Minns 2005). Our large‐scale models simulate and predict potential habitats of species that could be not locally realized habitat. Future studies could integrate local river physical, chemical, and biotic factors to derive a more realized habitat distribution. Despite this, SDMs still will have great potential for basin‐scale fish habitat simulations. Ensemble modeling of more machine‐learning algorithms could be used and species interaction could be incorporated in the model to provide more robust and reliable predictions in further work.

## 5 Conclusion

In this study, we explored using SDMs to simulate and predict the potential fish habitat distribution for two typical fish species in the UYRB. By using the implicit information of dam construction in the occurrence data, the impact of dam construction was analyzed based on modeling results of pre‐dam and post‐dam periods. The separate impact of climate change was disentangled based on future predictions under the combined impact of climate change and dam construction. Our findings indicate that dam construction has significantly reduced the suitable habitats for both warm‐water and cold‐water fish species. Although future climate change could lead to fish habitat expansion for the warm‐water fish, the *C. guichenoti*, the negative impact of dam construction was still predominant. What deserves more attention is that future climate change would exacerbate the dam's negative impact on the coldwater fish, *S. malacanthus*. The results of this study highlighted the importance of habitat conservation and restoration of endemic fish habitat in the UYRB under multiple changing environments. Despite certain limitations inherent in this study, the methods proposed herein for decomposing and quantifying the impacts of climate change and dam construction on fish habitats contribute to filling a gap in the fish habitat modeling and evaluation of large‐scale river systems. The research can serve as a scientific reference for scholars in habitat prediction of warm‐water fishes and cold‐water fishes for possible intercontinental and global work.

## Author Contributions

**Xiongfeng Bai:** data curation (lead), software (lead), visualization (lead), writing – original draft (lead), writing – review and editing (equal). **Peng Zhang:** conceptualization (lead), methodology (equal), supervision (lead), writing – review and editing (lead). **Xin Cao:** investigation (lead), software (equal). **Dongya Zhang:** data curation (supporting), investigation (supporting). **Zhi Yang:** data curation (supporting), investigation (supporting). **Xianghong Dong:** data curation (supporting), investigation (supporting). **Siyang Wang:** data curation (supporting), investigation (supporting). **Wenbin Li:** data curation (supporting), visualization (supporting). **Lihua Xiong:** funding acquisition (lead), project administration (lead).

## Conflicts of Interest

The authors declare no conflicts of interest.

## Supporting information


Appendix S1

## Data Availability

All fish occurrence data used in this paper are provided in Appendix S1. The environmental data used (except for River order) can be downloaded via the link in Appendix S3. The Env data (includes River order) for modeling and outputs of the model can be downloaded in https://datadryad.org/stash/share/SQtVuQi4YYVOyMhWNXhbLqX05sYmzqWGP3LC3W3vT6U.
