# Supplementary material for: Incorporating Implicit Information to Disentangle the Impacts of Hydropower Dams and Climate Change on Basin‐Scale Fish Habitat Distribution
Source: Ecol Evol. 2024 Oct 8;14(10):e70412. doi: 10.1002/ece3.70412 (PMC11461754; doi:10.1002/ece3.70412)
Supplement: Supplementary file 1 — Appendix S1 [file ECE3-14-e70412-s001.docx]

**S1** The Occurrence data for *Coreius guichenoti* and *Schizopygopsis malacanthus*

| Species name | Longitude | Latitude | Sources | Period |
| --- | --- | --- | --- | --- |
| CG | 100.3991928 | 26.30474091 | CAS | Pre-dam |
| CG | 101.6557465 | 26.58343315 | CAS | Pre-dam |
| CG | 101.699783 | 26.94993165 | CAS | Pre-dam |
| CG | 101.7652664 | 26.99317932 | CAS | Pre-dam |
| CG | 101.795517 | 26.81483269 | CAS | Pre-dam |
| CG | 101.88622 | 26.726416 | CAS | Pre-dam |
| CG | 101.9599178 | 27.70956381 | CAS | Pre-dam |
| CG | 104.160094 | 28.64125577 | CAS | Pre-dam |
| CG | 103.6535377 | 30.63217835 | GBIF^[1]^ | Pre-dam |
| CG | 103.7614212 | 29.54811859 | GBIF | Pre-dam |
| CG | 104.6338392 | 28.77424482 | GBIF | Pre-dam |
| CG | 104.6363907 | 28.76861382 | GBIF | Pre-dam |
| CG | 105.441803 | 28.89758492 | GBIF | Pre-dam |
| CG | 103.2146378 | 27.56278992 | Ding, 1994^[2]^ | Pre-dam |
| CG | 103.8699265 | 28.31505966 | Ding, 1994 | Pre-dam |
| CG | 104.1490631 | 29.05358315 | Ding, 1994 | Pre-dam |
| CG | 103.8870468 | 30.29436874 | Ding, 1994 | Pre-dam |
| CG | 104.6066988 | 28.60304907 | Ding, 1994 | Pre-dam |
| CG | 105.07217 | 29.039743 | Ding, 1994 | Pre-dam |
| CG | 106.1097488 | 29.13092995 | Ding, 1994 | Pre-dam |
| CG | 106.22434 | 29.923187 | Ding, 1994 | Pre-dam |
| CG | 106.2212067 | 30.41193581 | Ding, 1994 | Pre-dam |
| CG | 107.13224 | 29.832436 | Ding, 1994 | Pre-dam |
| CG | 107.8545227 | 30.06074333 | Ding, 1994 | Pre-dam |
| CG | 107.8478964 | 29.79685063 | Ding, 1994 | Pre-dam |
| CG | 108.3230362 | 30.6010952 | Ding, 1994 | Pre-dam |
| CG | 108.8218918 | 30.95248604 | Ding, 1994 | Pre-dam |
| CG | 109.213829 | 30.96581841 | Ding, 1994 | Pre-dam |
| CG | 109.6721573 | 31.0059433 | Ding, 1994 | Pre-dam |
| CG | 101.8355713 | 26.75984764 | CAS | Post-dam |
| CG | 101.8700532 | 28.46000771 | CAS | Post-dam |
| CG | 101.9600506 | 27.70956903 | CAS | Post-dam |
| CG | 101.7331696 | 26.59098625 | CAS | Post-dam |
| CG | 104.5220533 | 28.68684169 | CAS | Post-dam |
| CG | 100.4090773 | 26.650728 | CAS | Post-dam |
| CG | 100.7897755 | 26.19096099 | CAS | Post-dam |
| CG | 101.1815665 | 26.35755623 | CAS | Post-dam |
| CG | 101.6881714 | 26.56097603 | CAS | Post-dam |
| CG | 102.8738811 | 27.05167096 | CAS | Post-dam |
| CG | 103.7773438 | 28.25300217 | CAS | Post-dam |
| CG | 103.8784103 | 28.62539101 | CAS | Post-dam |
| CG | 104.4251121 | 28.62780244 | CAS | Post-dam |
| CG | 101.7998885 | 26.61072553 | CAS | Post-dam |
| CG | 100.3991928 | 26.30474091 | CAS | Post-dam |
| CG | 100.8598938 | 26.16592789 | CAS | Post-dam |
| CG | 101.2686996 | 26.36183929 | CAS | Post-dam |
| CG | 101.5439115 | 26.59419178 | CAS | Post-dam |
| CG | 102.8990418 | 26.90940247 | CAS | Post-dam |
| CG | 102.0688134 | 25.98026147 | CAS | Post-dam |
| CG | 101.5487302 | 26.59511637 | CAS | Post-dam |
| CG | 102.8095563 | 26.968813 | CAS | Post-dam |
| CG | 101.7496719 | 28.53013992 | CAS | Post-dam |
| CG | 101.4161911 | 26.39441872 | Tang et al., 2012^[3]^ | Post-dam |
| CG | 100.91639 | 26.070105 | Tang et al., 2012 | Post-dam |
| CG | 100.2943 | 25.825212 | Tang et al., 2012 | Post-dam |
| CG | 100.3942035 | 26.70356449 | He et al., 2022^[4]^ | Post-dam |
| CG | 100.4192658 | 26.49169497 | He et al., 2022 | Post-dam |
| CG | 102.8811289 | 27.28860523 | He et al., 2022 | Post-dam |
| CG | 103.870697 | 28.3210907 | He et al., 2022 | Post-dam |
| SM | 97.80095629 | 33.40948369 | Wu & Wu, 1990^[5]^ | Pre-dam |
| SM | 97.98553842 | 33.21269497 | Wu & Wu, 1990 | Pre-dam |
| SM | 98.22095616 | 33.11681346 | Wu & Wu, 1990 | Pre-dam |
| SM | 98.36235946 | 32.93298665 | Wu & Wu, 1990 | Pre-dam |
| SM | 98.63790477 | 32.86916177 | Wu & Wu, 1990 | Pre-dam |
| SM | 98.83425916 | 32.77998375 | Wu & Wu, 1990 | Pre-dam |
| SM | 99.06045287 | 32.69397461 | Wu & Wu, 1990 | Pre-dam |
| SM | 97.98220655 | 33.35654664 | Wu & Wu, 1990 | Pre-dam |
| SM | 98.31962468 | 33.17678736 | Wu & Wu, 1990 | Pre-dam |
| SM | 98.66348975 | 32.92567401 | Wu & Wu, 1990 | Pre-dam |
| SM | 101.470062 | 28.07379214 | Wu & Wu, 1986^[6]^ | Pre-dam |
| SM | 101.3827913 | 28.27111859 | Wu & Wu, 1986 | Pre-dam |
| SM | 101.069758 | 29.15866643 | Wu & Wu, 1986 | Pre-dam |
| SM | 101.0108838 | 30.20402069 | Wu & Wu, 1986 | Pre-dam |
| SM | 100.616782 | 30.39227429 | Wu & Wu, 1986 | Pre-dam |
| SM | 100.2334188 | 30.63751475 | Wu & Wu, 1986 | Pre-dam |
| SM | 98.20588097 | 33.11863072 | Wu & Wu, 1986 | Pre-dam |
| SM | 98.775162 | 32.237175 | Ding, 1994 | Pre-dam |
| SM | 99.46688843 | 32.03873062 | Ding, 1994 | Pre-dam |
| SM | 100.13163 | 30.052994 | Ding, 1994 | Pre-dam |
| SM | 100.89349 | 29.862247 | Ding, 1994 | Pre-dam |
| SM | 101.23716 | 31.074854 | Ding, 1994 | Pre-dam |
| SM | 101.503 | 29.711868 | Ding, 1994 | Pre-dam |
| SM | 102.70992 | 30.481253 | Ding, 1994 | Pre-dam |
| SM | 103.0082 | 30.772961 | Ding, 1994 | Pre-dam |
| SM | 104.06711 | 30.531889 | Ding, 1994 | Pre-dam |
| SM | 101.72517 | 30.761669 | Ding, 1994 | Pre-dam |
| SM | 101.75942 | 29.828796 | Ding, 1994 | Pre-dam |
| SM | 102.203375 | 28.62701577 | Ding, 1994 | Pre-dam |
| SM | 104.07353 | 30.575624 | Ding, 1994 | Pre-dam |
| SM | 96.496407 | 33.851433 | Ding, 1994 | Pre-dam |
| SM | 97.15682287 | 33.8094765 | Ding, 1994 | Pre-dam |
| SM | 95.81013489 | 34.0657959 | Ding, 1994 | Pre-dam |
| SM | 97.24636078 | 33.00503159 | Ding, 1994 | Pre-dam |
| SM | 102.1868151 | 28.52822508 | Ding, 1994 | Pre-dam |
| SM | 102.0688254 | 31.48394704 | Zhang et al., 2023^[7]^ | Post-dam |
| SM | 101.9940039 | 31.19072725 | Zhang et al., 2023 | Post-dam |
| SM | 101.881385 | 30.88856188 | Zhang et al., 2023 | Post-dam |
| SM | 100.6778154 | 31.39868139 | Zheng et al., 2016^[8]^ | Post-dam |
| SM | 100.7427095 | 31.32283609 | Zheng et al., 2016 | Post-dam |
| SM | 100.8073034 | 31.25437947 | Zheng et al., 2016 | Post-dam |
| SM | 96.54337785 | 33.194957 | Yang et al., 2022^[9]^ | Post-dam |
| SM | 97.21528182 | 32.99136883 | Yang et al., 2022 | Post-dam |
| SM | 96.91936692 | 32.86593545 | Yang et al., 2022 | Post-dam |
| SM | 99.06472222 | 29.93388889 | Xiong et al., 2022^[10]^ | Post-dam |
| SM | 99.06305556 | 29.95 | Xiong et al., 2022 | Post-dam |
| SM | 99.09083333 | 29.98305556 | Xiong et al., 2022 | Post-dam |
| SM | 99.1375 | 30.02916667 | Xiong et al., 2022 | Post-dam |
| SM | 99.15944444 | 30.06694444 | Xiong et al., 2022 | Post-dam |
| SM | 99.19583333 | 30.1625 | Xiong et al., 2022 | Post-dam |
| SM | 99.21722222 | 30.20722222 | Xiong et al., 2022 | Post-dam |
| SM | 97.9961494 | 32.45964534 | Hu et al., 2012^[11]^ | Post-dam |
| SM | 98.9626374 | 30.45456757 | Hu et al., 2012 | Post-dam |
| SM | 98.89425528 | 31.37835889 | Zhu et al., 2016^[12]^ | Post-dam |
| SM | 98.94144972 | 31.38532194 | Zhu et al., 2016 | Post-dam |
| SM | 99.16932583 | 31.33101722 | Zhu et al., 2016 | Post-dam |
| SM | 99.26789139 | 31.46450028 | Zhu et al., 2016 | Post-dam |
| SM | 99.57112806 | 31.26085556 | Zhu et al., 2016 | Post-dam |
| SM | 99.56973667 | 31.24722889 | Zhu et al., 2016 | Post-dam |
| SM | 99.56528556 | 31.20972639 | Zhu et al., 2016 | Post-dam |
| SM | 101.5243688 | 30.02485027 | Sun et al., 2022^[13]^ | Post-dam |
| SM | 101.5889895 | 29.94739517 | Sun et al., 2022 | Post-dam |
| SM | 101.4882269 | 29.69424402 | Sun et al., 2022 | Post-dam |
| SM | 101.3834571 | 29.64019034 | Sun et al., 2022 | Post-dam |
| SM | 97.11083333 | 33.36916667 | Qi et al., 2006^[14]^ | Post-dam |
| SM | 97.27760721 | 32.92276944 | Lei, 2020^[15]^ | Post-dam |
| SM | 96.80255267 | 33.35083923 | Lei, 2020 | Post-dam |
| SM | 96.65438124 | 33.53782031 | Lei, 2020 | Post-dam |
| SM | 100.0425358 | 29.84159969 | Shen et al., 2007^[16]^ | Post-dam |
| SM | 100.0901589 | 29.40027224 | Shen et al., 2007 | Post-dam |
| SM | 100.3884148 | 28.95793188 | Shen et al., 2007 | Post-dam |
| SM | 99.08917409 | 29.97854921 | Liu & Sun, 2017^[17]^ | Post-dam |
| SM | 99.08917409 | 30.2043965 | Liu & Sun, 2017 | Post-dam |
| SM | 102.1782273 | 28.41559771 | Ru et al., 2016^[18]^ | Post-dam |
| SM | 102.1947188 | 28.52432696 | Ru et al., 2016 | Post-dam |
| SM | 102.2087504 | 28.66787011 | Ru et al., 2016 | Post-dam |
| SM | 101.0997915 | 29.84175229 | Luo, 2019^[19]^ | Post-dam |
| SM | 101.0943548 | 29.803768 | Luo, 2019 | Post-dam |
| SM | 101.0958484 | 29.76342562 | Luo, 2019 | Post-dam |
| SM | 100.7791932 | 30.2197851 | Luo, 2019 | Post-dam |
| SM | 101.0091635 | 30.20403324 | Luo, 2019 | Post-dam |
| SM | 101.5345278 | 30.30791111 | Deng et al., 2022^[20]^ | Post-dam |
| SM | 101.5042722 | 30.137 | Deng et al., 2022 | Post-dam |
| SM | 101.56105 | 29.98794722 | Deng et al., 2022 | Post-dam |
| SM | 101.5579611 | 29.85523611 | Deng et al., 2022 | Post-dam |
| SM | 101.5183917 | 29.76169444 | Deng et al., 2022 | Post-dam |
| SM | 101.3694333 | 29.61946667 | Deng et al., 2022 | Post-dam |
| SM | 101.2994389 | 29.41978611 | Deng et al., 2022 | Post-dam |
| SM | 101.1851111 | 29.40595556 | Deng et al., 2022 | Post-dam |
| SM | 101.1530111 | 29.41795556 | Deng et al., 2022 | Post-dam |
| SM | 101.5137583 | 29.95188611 | Deng et al., 2022 | Post-dam |
| SM | 101.4312306 | 29.50744722 | Deng et al., 2022 | Post-dam |

CG and SM refers to *Coreius guichenoti* and *Schizopygopsis malacanthus*; CAS means Institute of Hydrobiology-Chinese Academy of Sciences; Pre-dam period corresponds to 1970-2000, post-dam corresponds to 2001-2020.


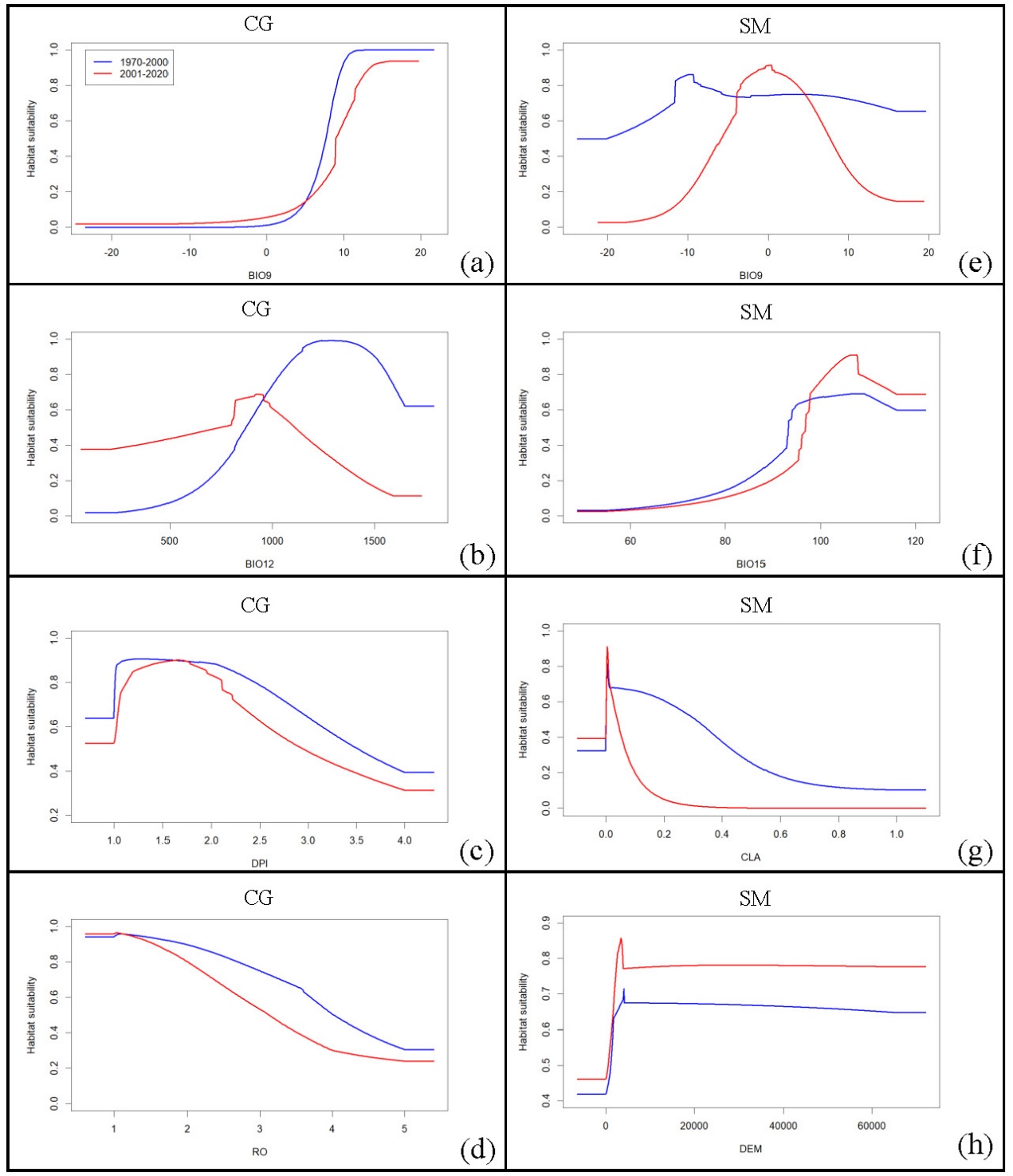


**S2** Average response curves of *C.guichenoti* (CG) and *S.malacanthus* (SM) models for the pre-dam (1970-2000) and post-dam (2001-2020) periods

**S3** The source of 4 human activity-related variables and 4 natural hydro-morphological variables

|  | Variables | Sources |
| --- | --- | --- |
| **Human activity** | CLA^[21-22]^ | https://sedac.ciesin.columbia.edu/data/set/aglands-croplands-2000 |
|  | DPI^[23-24]^ | https://sedac.ciesin.columbia.edu/data/set/lulc-development-threat-index |
|  | HIP^[25]^ | https://sedac.ciesin.columbia.edu/data/set/ulandsat-hbase-v1/data-download |
|  | POPC^[26]^ | https://sedac.ciesin.columbia.edu/data/set/gpw-v4-population-count-rev11/data-download |
| **hydro-morphology** | DEM | http://srtm.csi.cgiar.org/download |
|  | FHF^[27-28]^ | https://sedac.ciesin.columbia.edu/data/set/ndh-flood-hazard-frequency-distribution |
|  | RO | Self-made |
|  | WTA^[29]^ | https://sedac.ciesin.columbia.edu/data/set/gpw-v4-land-water-area-rev11 |

**S4** GCMs and Institution for BIO^[30]^

| GCMs | Institution |
| --- | --- |
| CNRM-ESM2-1 | Centre National de Recherches Météorologiques |
| MIROC-ES2L | (1) Atmosphere and Ocean Research Institute  (2) National Institute for Environmental Studies  (3) Japan Agency for Marine-Earth Science and Technology |


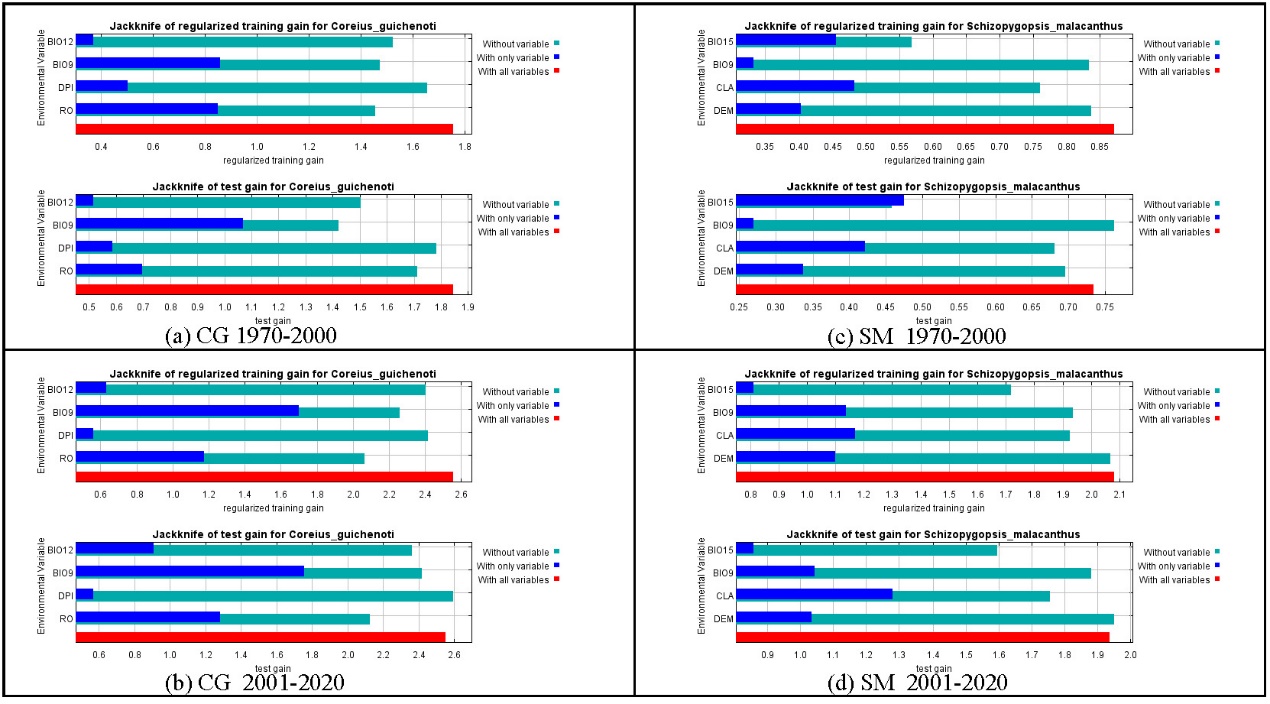


**S5**: Mean variable importance to the habitat distribution of *C.guichenoti* (CG) and *S.malacanthus* (SM) determined by jackknife-cut method for the pre-dam (1970-2000) and post-dam (2001-2020) periods. They were both affiliated with training and [testing dataset](https://www.sciencedirect.com/topics/engineering/testing-dataset), respectively.


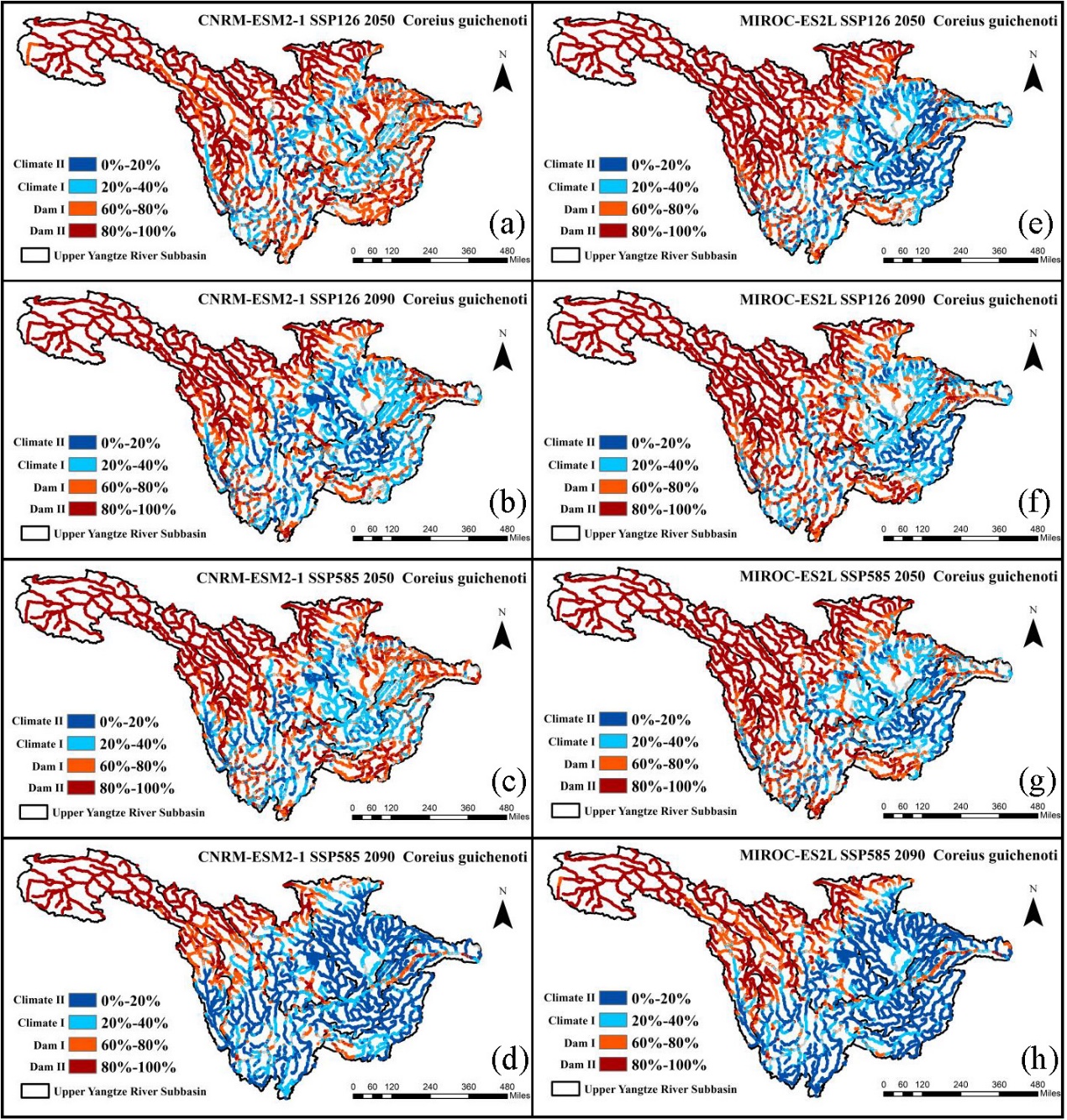


**S6** $I_{\text{e}}$ of *C.guichenoti* (CG). Climate II and Climate I corresponds to C2 and C 1, Dam II and Dam I corresponds to D2 and D1.


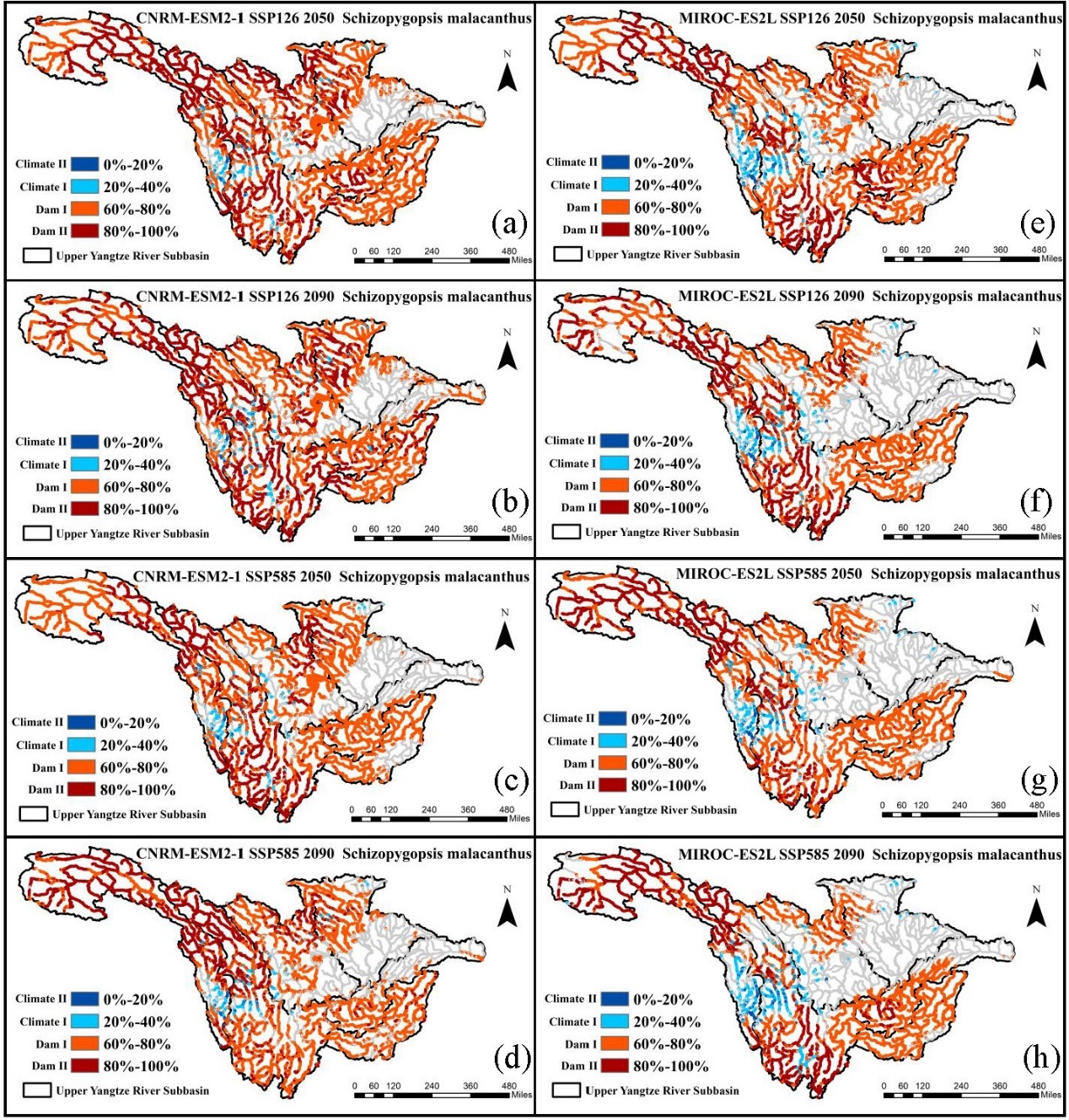


**S7** $I_{\text{e}}$ of *S.malacanthus* (SM). Climate II and Climate I corresponds to C2 and C 1, Dam II and Dam I corresponds to D2 and D1.

**References**

[1] GBIF.org (12 April 2024) GBIF Occurrence Download. [https://doi.org/10.15468/dl.qxu88q](%20https:/doi.org/10.15468/dl.qxu88q)

[2] Ding, D. (1994). Sichuan Fish Records (In Chinese). *Chengdu: Sichuan Science and Technology Press*.

[3] Tang, H., Yang, Z., Gao, S., Chen, J., Zhang, Y., Wan, L., & Qiao, Y. (2012). Status of Fish Resources of Early Life History Stages of Coreius guichenoti in the Middle Reaches of the Jinsha River (In Chinese). *Sichuan Journal of Zoology*, 31(3), 416-421. <https://doi.org/10.3969/j.issn.1000-7083.2012.03.017>

[4] He, Y., Zhu, Y., Gong, J., Zhu, T., Wu, X., Li, X., Meng, Z., & Yang, D. (2022). Genetic Diversity and Population Demography of *Coreius guichenoti* from the Middle and Lower Reaches of the Jinsha River (In Chinese). *Acta Hydrobiologica Sinica*, 46(1), 37-47. <https://doi.org/10.7541/2021.2020.255>

[5] Wu, J., & Wu, M. (1990). The fish fauna of the Jinsha River (In Chinese). *Sichuan Journal of Zoology*, 9(3), 23-26.

[6] Wu, J., & Wu, M. (1986). Natural fishery resources of the Yalong River (In Chinese). *Sichuan Journal of Zoology*, (01), 1-5, 10.

[7] Zhang, Z., Liang, W., Zhang, D., Zhou, W., Yang, Y., Cheng, R., Xiong, W., Chang, X., & Chen, F. (2023). Fish Resources and Diversity in the Mainstem and Tributaries of Dadu River from Jinchuan to Danba (In Chinese). *Journal of Hydroecology*, 44(3), 54-61. <https://doi.org/10.15928/j.1674-3075.202206060215>

[8] Zheng, C., Wu, Y., & Sun, Z. (2016). The Present Situation of Wetland Resources in Huoerzhanggu National Wetland Park and Their Evaluation (In Chinese). *Journal of Sichuan Forestry Science and Technology*, 37(6), 38-44, 58. <https://doi.org/10.16779/j.cnki.1003-5508.2016.06.008>

[9] Yang, C., Gao, Q., Liu, D., Nie, M., Wang, F., Li, K., Zhao, H., Zhang, C., & Qi, D. (2022). Genetic Diversity of *Schizopygopsis malacanthus* Based on Partial Sequence of mtDNA D-loop (In Chinese). *Sichuan Journal of Zoology*, 41(04), 398-405. <https://doi.org/10.11984/j.issn.1000-7083.20210445>

[10] Xiong, M., Shao, K., Dong, W., Chen, H., Zeng, C., Que, Y., Chen, F., & Zhu, B. (2022). Study on Fish Community Structure of the Main River below Batang Hydropower Station and the Tributary Bachu River in the Upper Reaches of the Jinsha River (In Chinese). *Resources and Environment in the Yangtze Basin*, 31(11), 2481-2488. <https://doi.org/10.11870/cjlyzyyhj202211013>

[11] Hu, R., Wang, J., Tan, D., Miao, Z., & Dan, S. (2012). Age and Growth of *Schizopygopsis malacanthus* Herzenstein in the Upper Reaches of the Chin-sha River (In Chinese). *Sichuan Journal of Zoology*, 31(05), 708-712, 719, 849. <https://doi.org/10.3969/j.issn.1000-7083.2012.05.004>

[12] Zhu, T., Li, F., & Yang, D. (2016). Fish Resources and Feeding Habits of *Ptychobarbus Kaznakovi* in the Zengqu River, a Tributary of the Upper Jinsha River (In Chinese). *Resources and Environment in the Yangtze Basin*, 25(07), 1086-1092. <https://doi.org/10.11870/cjlyzyyhj201607010>

[13] Sun, G., Luo, Z., Zhao, Y., Han, R., & Xiao, N. (2022). Field Investigation and Evaluation of Fish in Kangding City (In Chinese). *Sichuan Journal of Zoology*, 41(01), 83-91. <https://doi.org/10.11984/j.issn.1000-7083.20210174>

[14] Qi, D., Guo, S., & Zhao, X. (2006). Molecular systematics of two enigmatic fishes in the genus *Schizopygopsis* in the Qinghai-Tibetan Plateau (In Chinese). *Acta Zoologica Sinica*, (06), 1058-1066.

[15] Lei, M. (2021). Investigation on Helminth Fauna of Fish in Qinghai and Phylogenetic Analysis of *Echinorhynchus gymnocyprii* (In Chinese). *Doctor dissertation, Northwest A&F University*. <https://doi.org/10.27409/d.cnki.gxbnu.2020.001449>

[16] Shen, D., He, C., & Song, Z. (2007). Age Determination of Weakspine Schizothoracin *(Schizopygopsis malacanthus)* (In Chinese)*.* *Sichuan Journal of Zoology*, (01), 124-125, 241.

[17] Liu, R., & Sun, Y. (2017). Study on the habitat protection model of tributary fish habitats in hydropower development (In Chinese). *Environment and Development*, 29(08), 211-213. <https://doi.org/10.16647/j.cnki.cn15-1369/X.2017.08.124>

[18] Ru, H., Zhang, Y., Li, Y., Wang, H., Shen, Z., Wu, X., Li, R., Sheng, Q., & Ni, C. (2016). Community Composition and Status of Fish Resources in Anning River (In Chinese). *Journal of Hydroecology*, 37(05), 68-74. <https://doi.org/10.15928/j.1674-3075.2016.05.010>

[19] Luo, J. (2019). Fish community structure and growth characteristics and genetic structure analysis of *Schizothorax prenanti* in middle reaches of Yalong River (In Chinese). *Master dissertation, Sichuan Agricultural University*. <https://doi.org/10.27345/d.cnki.gsnyu.2019.001464>

[20] Deng, J., Wang, X., Tong, L., Xu, L., Xiang, P., Sun, G., Xiao, N., & Song, Z. (2022). Fish Diversity and Spatial Pattern in the Liqiu River, a Tributary of the Middle Yalong River (In Chinese). *Sichuan Journal of Zoology*, 41(04), 444-453. <https://doi.org/10.11984/j.issn.1000-7083.20220015>

[21] [Dataset] Ramankutty, N., A.T. Evan, C. Monfreda, and J.A. Foley. 2010. Global Agricultural Lands: Croplands, 2000. Palisades, New York: NASA Socioeconomic Data and Applications Center (SEDAC). <https://doi.org/10.7927/H4C8276G>

[22] Ramankutty, N., A.T. Evan, C. Monfreda, and J.A. Foley. 2008. Farming the Planet: 1. Geographic Distribution of Global Agricultural Lands in the Year 2000. *Global Biogeochem. Cycles 22 (1): GB1003*. <https://doi.org/10.1029/2007GB002952>

[23] [Dataset] Oakleaf, J. R., C. M. Kennedy, S. Baruch-Mordo, P. C. West, J. S. Gerber, L. Jarvis, and J. Kiesecker. 2019. Development Threat Index. Palisades, New York: NASA Socioeconomic Data and Applications Center (SEDAC). <https://doi.org/10.7927/61jv-th84>

[24] Oakleaf, J. R., C. M. Kennedy, S. Baruch-Mordo, P. C. West, J. S. Gerber, L. Jarvis and J. Kiesecker. 2015. A World at Risk: Aggregating Development Trends to Forecast Global Habitat Conversion. *PLoS ONE* 10(10): e0138334. <https://doi.org/10.1371/journal.pone.0138334>

[25] [Dataset] Wang, P., C. Huang, E. C. Brown de Colstoun, J. C. Tilton, and B. Tan. 2017. Global Human Built-up And Settlement Extent (HBASE) Dataset From Landsat. Palisades, New York: NASA Socioeconomic Data and Applications Center (SEDAC). <https://doi.org/10.7927/H4DN434S>

[26] [Dataset] Center for International Earth Science Information Network - CIESIN - Columbia University. 2018. Gridded Population of the World, Version 4 (GPWv4): Population Count, Revision 11. Palisades, New York: NASA Socioeconomic Data and Applications Center (SEDAC).  <https://doi.org/10.7927/H4JW8BX5>

[27] [Dataset] Center for Hazards and Risk Research - CHRR - Columbia University, and Center for International Earth Science Information Network - CIESIN - Columbia University. 2005. Global Flood Hazard Frequency and Distribution. Palisades, New York: NASA Socioeconomic Data and Applications Center (SEDAC). <https://doi.org/10.7927/H4668B3D>

[28] Dilley, M., R.S. Chen, U. Deichmann, A.L. Lerner-Lam, M. Arnold, J. Agwe, P. Buys, O. Kjekstad, B. Lyon, and G. Yetman. 2005. Natural Disaster Hotspots: A Global Risk Analysis. *Washington, D.C.: World Bank*.[http://documents.worldbank.org/curated/en/621711468175150317/Natural-disaster-hotspots-A-global-risk-analysis](file:///C:\Users\hp\Desktop\SCI1\9_version9_zp\ http:\documents.worldbank.org\curated\en\621711468175150317\Natural-disaster-hotspots-A-global-risk-analysis)

[29] [Dataset] Center for International Earth Science Information Network - CIESIN - Columbia University. 2018. Gridded Population of the World, Version 4 (GPWv4): Land and Water Area, Revision 11. Palisades, New York: NASA Socioeconomic Data and Applications Center (SEDAC). <https://doi.org/10.7927/H4Z60M4Z>

[30] Eyring, V., Bony, S., Meehl, G. A., Senior, C. A., Stevens, B., Stouffer, R. J., & Taylor, K. E. (2016). Overview of the Coupled Model Intercomparison Project Phase 6 (CMIP6) experimental design and organization[Dataset]. *Geoscientific Model Development*, *9*(5), 1937-1958. <https://doi.org/10.5194/gmd-9-1937-2016>
